# Heterologous expression of diverse propionyl-CoA carboxylases affects polyketide production in *Escherichia coli*

**DOI:** 10.1038/ja.2017.38

**Published:** 2017-04-12

**Authors:** Gergana A Vandova, Robert V O'Brien, Brian Lowry, Thomas F Robbins, Curt R Fischer, Ronald W Davis, Chaitan Khosla, Colin JB Harvey, Maureen E Hillenmeyer

**Affiliations:** 1 Stanford Genome Technology Center, Stanford University, Palo Alto, CA, USA; 2 Department of Biochemistry, Stanford University, Palo Alto, CA, USA; 3 Department of Chemistry, Stanford University, Stanford, CA,USA; 4 Department of Chemical Engineering, Stanford University, Stanford, CA, USA; 5 Stanford ChEM-H Institute, Stanford University, Stanford, CA, USA

## Introduction

Polyketide synthase (PKS) enzymes catalyze the biosynthesis of polyketide natural products.^1^ One class of PKS enzymes, called multi-modular or assembly-line PKSs, are organized into multiple modules, where each module covalently adds a ketide unit derived from an acyl-CoA to the growing natural product. PKSs of this class produce the polyketide backbone of antibacterials (erythromycin), antifungals (amphotericin), immunosuppressive agents (rapamycin and FK506) and anticancer compounds (epothilone). In microbial genomes, PKS genes are often co-localized with all other genes required for the production of a given compound in a biosynthetic gene cluster. With the increasing ease of DNA sequencing, hundreds of novel assembly-line PKS gene clusters have been identified in recent years.^2, 3^

Heterologous expression of natural product genes in model hosts has been applied to access novel natural products including polyketides.^4^ *Escherichia coli* is an attractive host for many reasons: it is easy to culture with a well-developed genetic toolbox, the primary metabolism is well understood, and because it is not an endogenous producer of polyketides, potential interference of native proteins with heterologously expressed PKS pathways may be limited.^5^ Despite these advantages, attempts at heterologous production of polyketides in *E. coli* have met with limited success. Examples of assembly-line PKS pathways expressed in *E. coli* include those encoding epothilone^6^ and 6-deoxyerythronolide B (6-dEB), the precursor to erythromycin.^7^ Titers for 6-dEB have ranged from 1 to 20 mg l^−1^, whereas titers for epothilone were significantly lower (0.001 mg l^−1^). These low titers have limited the utility of *E. coli* as a host for production of assembly-line polyketides, and have spurred efforts to improve general characteristics of this host to produce this class of compounds.^7^

These limitations point to a need for systematic optimization of heterologously expressed pathways. One approach to improving *E. coli* as a host has been to increase availability of the substrates of PKS enzymes.^8^ For many assembly-line PKS pathways, the extender unit for the polyketide chain is (2*S*)-methylmalonyl-CoA (mmCoA), a metabolite not naturally produced by *E. coli.*^9^ Murli *et al.*^10^ compared three routes of heterologous mmCoA production in *E. coli*: (1) carboxylation of propionyl-CoA by the propionyl-CoA carboxylase (PCC) from *Streptomyces coelicolor*, (2) conversion of succinyl-CoA to (2*R*)-mmCoA and then to (2*S*)-mmCoA by the mmCoA mutase/epimerase from *Propionibacterium shermanii* and (3) synthesis of (2*S*)-mmCoA from methylmalonate by the malonyl/methylmalonyl-CoA ligase from *S. coelicolor*. The 6-dEB titers varied depending on the means of mmCoA production, with the PCC system consistently proving the most productive. These results suggested that, despite the low turnover rate of the 6-deoxyerythronolide B synthase (DEBS),^11^ the choice of pathway for heterologous mmCoA production has an effect on downstream polyketide titers. We hypothesized that further optimization of this upstream step, particularly of the promising PCC pathway, might further increase titers.

PCC is a biotin-dependent enzyme that catalyzes the carboxylation of propionyl-CoA to (2*S*)-mmCoA. In actinomycetes, such as *S. coelicolor,* the core catalytic PCC complex consists of α and β-subunits, each encoded by a distinct gene. The α-subunit consists of biotin carboxyl carrier protein and biotin carboxylase domains while the β-subunit has carboxyltransferase activity. Structural studies in *S. coelicolor* demonstrated that the β-subunits are homohexamers and implied an α_6_β_6_ PCC complex architecture.^12^

In this study, we examined the effect of PCC expression and identity on polyketide production in *E. coli* by both optimizing the expression of *S. coelicolor* PCC complex and screening of 13 homologous PCCs from diverse species.

## Results and discussion

**Figure 1:**
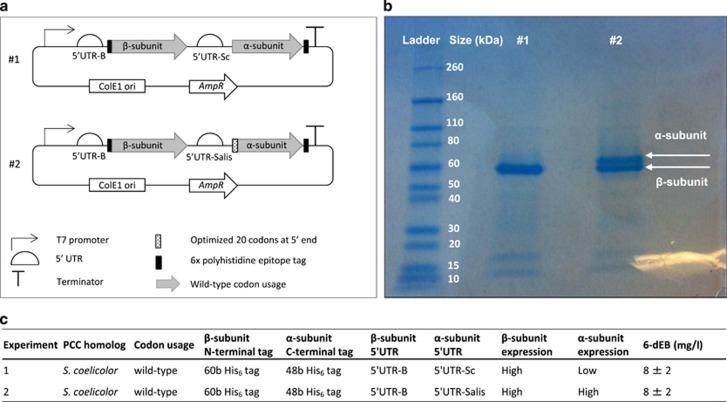
Propionyl-CoA carboxylase (PCC) protein expression and 6-dEB production of strains harboring wild type and optimized *S. coelicolor* PCC complexes. (**a**) Scheme representation of the PCC complex-harboring vectors. (**b**) Coomassie-stained denaturing protein gel of α and β-subunits of the wild type and computationally optimized *S. coelicolor* PCC constructs in the soluble cell lysate fraction. (**c**) Table of genetic design differences, PCC subunits expression, and 6-dEB production from the wild type and optimized constructs. 5′UTR, 5′ untranslated region; 6-dEB, 6-deoxyerythronolode B; AmpR, ampicillin resistance gene; ColE1 ori, colicin E1 origin of replication; His_6_ tag, 6x polyhistidine tag; PCC, propionyl-CoA carboxylase.

Our study of the effect of the PCC complex on heterologous polyketide production in *E. coli* began with a previously reported system in which heterologous expression of the DEBS genes along with the two genes comprising the *S. coelicolor* PCC complex (PCC α and PCC β-subunit)^7^ led to production of 6-dEB, the aglycone precursor of erythromycin. In this system, we observed poor expression of the *S. coelicolor* PCC α-subunit (Figure 1). Because a previous study showed that reduced expression of PCC subunits was correlated with reduced 6-dEB production,^13^ we first sought to improve PCC α-subunit protein expression. We applied three strategies to increase protein expression. First, we substituted the wild-type ribosome-binding site (RBS) sequence with the RBS sequence upstream of the PCC β-subunit, because the PCC β-subunit was highly expressed (Figure 1b, construct #1). Second, we optimized the first 20 codons to match the codon frequencies of *E. coli*, since rare codons in the 5′ end of the gene have been shown to reduce expression levels.^14^ Third, we applied a computational tool developed by Salis *et al.*^15^ to optimize this gene’s 5′ untranslated region (5′UTR) pre-RBS sequence upstream of the newly substituted RBS. This method correctly predicted low expression of the wild-type PCC α-subunit. Combining these three changes resulted in an optimized construct (Figure 1a, construct #2) that led to ~25-fold increase in α-subunit expression relative to the wild-type α-subunit expression (Figure 1b). Western blotting with streptavidin showed that the optimized α-subunit was biotinylated, whereas no biotinylation was detected on the wild-type α-subunit (Supplementary Figure S1). Notably, increased protein expression of the optimized PCC complex did not lead to increased polyketide production, when expressed in conjunction with the DEBS PKS genes (Figure 1c), suggesting that α-subunit expression was not limiting the ability of the *S. coelicolor* PCC complex to facilitate 6-dEB production in *E. coli*.

Since higher protein expression of the *S. coelicolor* PCC α-subunit did not result in higher polyketide titers, we turned our attention to other avenues for polyketide production improvement via PCC complexes. With the increasing availability of DNA sequence and synthesis, one strategy employed in metabolic engineering is comparison of homologous enzymes from diverse organisms.^16, 17, 18^ We hypothesized that homologous PCC complexes from nature could lead to higher polyketide titers than the single tested PCC complex from *S. coelicolor*, for example through higher specific PCC activity in *E. coli,* improved protein folding in *E. coli*, interaction with host factors necessary for enzymatic activity, or effects on the PKS itself. We selected genes encoding 13 PCC complexes from bacteria and eukaryotes, nine of which had experimentally verified PCC activity in the literature, and four of which were uncharacterized but shared sequence similarity with a verified PCC (Materials and Methods, Supplementary Figure S2, Supplementary Table S2).

**Figure 2:**
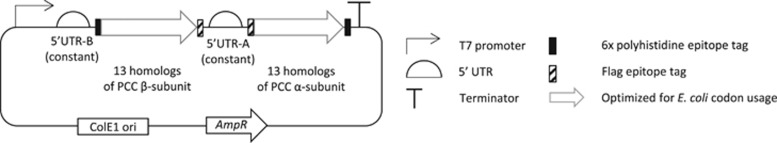
Scheme of genetic constructs of 13 engineered propionyl-CoA carboxylase complexes. 5′UTR, 5′ untranslated region; AmpR, ampicillin resistance gene; ColE1 ori, colicin E1 origin of replication; PCC, propionyl-CoA carboxylase.

To design PCC expression constructs, we began with the previously published PCC construct discussed above, referred to hereafter as the ‘wild-type’ PCC from *S. coelicolor*. In this design, one T7 promoter drives expression of the two *pcc* genes in *E. coli.*^7, 19, 20^ The β-subunit harbors an N-terminal polyhistidine tag (His_6_ tag). To facilitate standardized DNA synthesis and to assay protein expression for all PCC complex homologs, we made three major changes from the initial ‘wild-type’ design. First, each gene was flanked by an N-terminal His_6_ tag and a C-terminal Flag tag (Figure 2). Second, each α and β-subunit was preceded by a constant 5′UTR (named 5′UTR-A and 5′UTR-B, respectively), each harboring a distinct RBS to drive protein translation. Finally, as these genes come from diverse species, all genes, including those from *S. coelicolor*, were codon-optimized to match the codon frequencies of highly expressed *E. coli* genes through synonymous mutations.

We generated strains of *E. coli* that harbored an engineered PCC complex (two genes) on one plasmid (Figure 2), and the 6-dEB PKS pathway (three genes) on a second plasmid. Despite the relatively low maximum turnover rate of the complete hexamodular DEBS system *in vitro* (1.1 min^−1^ ; ref. 11), titers of 6-dEB ranged from 0.2 to 6 mg l^−1^ (Table 1) demonstrating that the identity of PCC significantly affects 6-dEB titers. Possible explanations include (1) *in vivo* mmCoA concentrations are below the saturation level of the DEBS system, and these concentrations are dependent on PCC identity, possibly through expression level, solubility, α-subunit biotinylation or activity, or (2) PCC identity affects DEBS protein expression. Measuring mmCoA concentrations and DEBS protein expression may clarify among these and other possible explanations.

The strain harboring a PCC homolog from *Myxococcus fulvus* produced the highest titer of 6-dEB among the 13 strains tested, despite low levels of PCC protein expression (Supplementary Figure S3). The strain harboring the engineered PCC homolog from *S. coelicolor* (the wild-type version of which had been previously studied) produced the third-highest titer. Four PCC pathways have been previously characterized in terms of kinetic parameters, but there was no observable trend between Km and 6-dEB production (Table S5).

We compared the *E. coli* strains harboring the 13 engineered PCCs to a strain harboring the previously tested wild-type *S. coelicolor* PCC (Supplementary Figure S4).^7, 19, 20^ To our surprise, the strain harboring the wild-type *S. coelicolor* PCC resulted in significantly higher titers (20 mg l^−1^) than all 13 strains harboring engineered PCC homologous complexes, including that of the engineered *S. coelicolor* PCC (3 mg l^−1^) and even the best-performing engineered homolog from *M. fulvus* (6 mg l^−1^, Table 2, experiments 1, 3 and 5).

This large difference in titer between the wild-type *S. coelicolor* PCC construct and all engineered PCC constructs prompted us to investigate the genetic differences that could be causing the titer differences. As described above, the engineered PCC constructs were modified from the wild-type constructs in three ways: (1) epitope tags were added to the engineered PCC subunits, (2) the 5′UTR upstream of each PCC subunit was modified, and (3) synonymous mutations of the *pcc* genes were engineered to match *E. coli* codon usage.

Since the PCC complex has an α_6_β_6_ architecture in several organisms, including species of *Streptomyces*^21^ and *Myxococcus,*^22^ we first considered the possibility that epitope tags could influence the protein complex in terms of stability or activity. To test the hypothesis that epitope tags on PCC proteins reduced polyketide titers, we built several new strains harboring engineered *S. coelicolor* and *M. fulvus* PCC complexes with presence and absence of N- and C-terminal epitope tags (Table 2). Eliminating two of the four epitope tags from the two PCC proteins dramatically increased polyketide production in strains expressing codon-optimized PCCs from both *S. coelicolor* (20 mg l^−1^ vs 3 mg l^−1^) and *M. fulvus* (10.5 mg l^−1^ vs 6 mg l^−1^, Table 2, experiments 3–6). Similarly, addition of a C-terminal His_6_ tag to the α-subunit of the wild-type *S. coelicolor* PCC significantly reduced polyketide titers (8 mg l^−1^ with both N- and C-terminal tags vs 20 mg l^−1^ with only the N-terminal tag, Table 2, experiments 2 and 1). When two of the four tags were eliminated, the engineered *S. coelicolor* homolog outperformed the engineered *M. fulvus* homolog (20 mg l^−1^ vs 10.5 mg l^−1^, Table 2, experiments 4 and 6) and matched the wild-type *S. coelicolor* PCC (20 mg l^−1^ vs 20 mg l^−1^, Table 2, experiments 4 and 1, respectively). These results demonstrate that certain epitope tags on PCC subunits substantially decrease polyketide production in *E. coli*.

The PCC complex is homologous to the acetyl-CoA carboxylase (ACC) complex, which exists natively in all bacteria, including *E. coli*. ACC catalyzes the carboxylation of acetyl-CoA to malonyl-CoA, which is the first committed step of fatty-acid biosynthesis. Similar to the PCC from *S. coelicolor*, the *E. coli* ACC is a multi-subunit complex, encoded by a biotin carboxylase, a biotin carboxyl carrier protein and a two-subunit carboxyltransferase.^23^ In *S. coelicolor*, the PCC and ACC complexes share a single α-subunit.^24^ In other species, the carboxylation of propionyl-CoA and acetyl-CoA are catalyzed by a single enzyme.^25, 26, 27, 28^ Since increased protein expression of the *S. coelicolor* α-subunit did not result in increased polyketide production (Figure 1), it is possible that either the *S. coelicolor* α-subunit is not rate-limiting, or the heterologous α-subunit is not required for 6-dEB production because the *E. coli* biotin carboxyl carrier protein subunit of ACC collaborates with the *S. coelicolor* β-subunit of PCC. We tested this hypothesis by measuring polyketide production in *E. coli* strains harboring the engineered *S. coelicolor* α and β-subunit individually. The strain expressing only the β-subunit in the absence of the cognate α-subunit produced similar titers to the strain harboring both subunits (4 mg l^−1^ vs 3 mg l^−1^, Table 2, experiments 8 and 3), suggesting that the native *E. coli* biotin carboxyl carrier protein subunit of ACC collaborates with the heterologously expressed *S. coelicolor* β-subunit of PCC. Also of note is that strains harboring the α-subunit but lacking the β-subunit produced significantly more 6-dEB than strains lacking both subunits (2.2 mg l^−1^ vs 0.5 mg l^−1^, Table 2, experiments 7 and 9), which could be explained by a modest PCC activity of the native *E. coli* carboxyltransferase subunit of ACC.

In conclusion, we investigated the effect of expressing 13 heterologous PCC complexes from different species on polyketide production in *E. coli*. We determined that variations in the PCC complex led to variation in polyketide production, suggesting that the PCC complex is, in some contexts, rate-limiting. This may be due to different activities of individual PCC enzymes when expressed in *E. coli*, differences in their solubility or biotinylation, or due to an effect on DEBS protein expression. Future work quantifying mmCoA and protein expression may determine the cause of the variability. We identified a PCC complex from the myxobacterium *M. fulvus* that outperformed all engineered PCC complexes in terms of polyketide production. We characterized the best-performing PCC homologs in *S. coelicolor* and *M. fulvus*, determining that the epitope tags drastically reduce polyketide production. This work demonstrates that, of the systems tested, the PCC complex from *S. coelicolor* supports the highest levels of heterologous polyketide production. The molar yield of 6-dEB of the *E. coli* strain, harboring the wild-type *S. coelicolor* PCC, which produced the highest 6-dEB titer, is 1.2%, which is in good agreement with what has been previously reported in literature.^29^ However, using flux balance analysis, the maximum theoretical molar yield of 6-dEB was calculated to be 10.7%.^29^ Given the poor-expression level and relatively high titer, it is possible that optimization of the *M. fulvus* PCC subunits, including eliminating all epitope tags, or by co-expressing additional open reading frames (Supplementary Figure S5), could lead to a superior polyketide production. Further improvement may be achieved through screening additional PCC homologous pathways in nature, or further engineering of the existing pathways.

## Figures and Tables

**Table 1:** 6-dEB production among 13 strains of *E. c* *oli* harboring heterologous PCC complexes from different species.

*Experiment*	*PCC homolog*	*6-dEB (mg l^–1^*)
1	*Myxococcus fulvus*	6±1
2	*Corynebacterium glutamicum*	4±2
3	*Streptomyces coelicolor*	3.0±0.4
4	*Actinoplanes sp.*	3±1
5	*Methylobacterium extorquens*	2.1±0.1
6	*Rhodobacter sphaeroides*	1.9±0.6
7	*Propionibacterium acnes*	1.7±0.5
8	*Danio rerio*	1.7±0.2
9	*Sinorhizobium fredii*	1.4±0.4
10	*Bos taurus*	1.3±0.2
11	*Paracoccus sp.*	0.6±0.2
12	*Homo sapiens*	0.4±0.2
13	*Chloroflexus aurantiacus*	0.2±0.1
14	No PCC	0.5±0.3

Abbreviations: 6-dEB, 6-deoxyerythronolide B; PCC, propionyl-CoA carboxylase.

Mean of three biological replicates±s.e.

**Table 2:** 6-dEB production in wild-type *S. coelicolor* PCC and engineered *S. coelicolor* and *M. fulvus* PCC strains.

*Experiment*	*PCC homolog*	*β-subunit present*	*α-subunit present*	*Codon usage*	*β-subunit 5*′*UTR*	*β-subunit N-terminal tag*	*β-subunit C-terminal tag*	*α-subunit 5*′*UTR*	*α-subunit N-terminal tag*	*α-subunit C-terminal tag*	*6-dEB (mg* *l^–1^)*
1	*S. coelicolor*	Yes	Yes	wild-type	5′UTR-B	N1	—	5′UTR-Sc	—	—	20 ± 3
2	*S. coelicolor*	Yes	Yes	wild-type	5′UTR-B	N1	—	5′UTR-Sc	—	C2	8±2
3	*S. coelicolor*	Yes	Yes	engineered	5′UTR-B	N2	C1	5′UTR-A	N3	C3	3.0±0.4
*4*	*S. coelicolor*	Yes	Yes	engineered	5′UTR-B	N2	—	5′UTR-A	—	C3	20±2
5	*M. fulvus*	Yes	Yes	engineered	5′UTR-B	N2	C1	5′UTR-A	N3	C3	6±1
6	*M. fulvus*	Yes	Yes	engineered	5′UTR-B	N2	—	5′UTR-A	—	C3	10.5±0.2
7	*S. coelicolor*	No	Yes	engineered	—	—	—	5′UTR-A	N3	C3	2.2± 0.7
8	*S. coelicolor*	Yes	No	engineered	5′UTR-B	N2	C1	—	—	—	4±1
9	No PCC	No	No	—	—	—	—	—	—	—	0.5±0.3

Abbreviations: PCC, propionyl-CoA carboxylase; 5′UTR, 5′ untranslated region.

N1-60b His_6_ tag, N2-42b His_6_ tag, C1-42b Flag tag, N3-42b Flag tag, C2-48b His_6_ tag, C3-42b His_6_ tag. Mean of three biological replicates±s.e.
