# Supplementary material for: Heterologous expression of diverse propionyl-CoA carboxylases affects polyketide production in Escherichia coli
Source: J Antibiot (Tokyo). 2017 Apr 12;70(7):859–63. doi: 10.1038/ja.2017.38 (PMC5509990; doi:10.1038/ja.2017.38)
Supplement: Supplementary Information [file ja201738x1.docx]

**Supplementary information**

**Heterologous expression of diverse propionyl-CoA carboxylases affects polyketide production in *Escherichia coli***

Gergana A. Vandova^1,2^, Robert V. O'Brien^4^, Brian Lowry^3^, Thomas F. Robbins^4^, Curt R. Fischer^1,5^ Ronald W. Davis^1,2^, Chaitan Khosla^3,4,5^, Colin J.B. Harvey^1^, and Maureen E. Hillenmeyer^1^,

^1^Stanford Genome Technology Center, Stanford University, Palo Alto, CA 94304;

^2^Department of Biochemistry, Stanford University, Palo Alto, CA 94305;

^3^Department of Chemical Engineering, Stanford University, Stanford, CA 94305;

^4^Department of Chemistry, Stanford University, Stanford, CA 94305;

^5^Stanford ChEM-H Institute, Stanford University, Stanford, CA 94305.

**Table of Contents**

Materials and Methods 3

Materials and Laboratory Services 3

Bacterial Strains 3

Selection of pcc genes for heterologous expression 3

Construction of PCC plasmids 4

Construction of MH109 5

Construction of second-generation PCC plasmids 5

Quantification of 6-dEB production using a liquid chromatography/mass spectrometry (LC/MS) assay 6

Protein Expression of PCC subunits 7

Western blotting of α-subunits 7

Supplementary Figures 9

Figure S1. Protein expression of α and β-subunits of the wild-type and computationally-optimized S. coelicolor PCC constructs (construct #1 and #2 from Figure 1). 9

Figure S2. (A) Phylogenetic tree of 13 PCC α-subunits and E. coli ACC BC subunit and (B) phylogenetic tree of 13 PCC β-subunits and E. coli ACC CT subunit. 10

Figure S3. Protein expression of engineered PCC α and β-subunits homologs. 11

Figure S4. Scheme of wild-type S. coelicolor PCC. 13

Figure S5. Graphic display of the PCC genome region of M. fulvus. 14

Figure S6. LC/MS analysis of authentic standard 6-dEB. 15

Figure S7. LC/MS standard curves of an authentic standard of 6-dEB run on both days LC/MS data was collected. 16

Supplementary Tables 17

Table S1. Plasmids used in this study. 17

Table S2. List of PCC enzyme homologs used in study. 18

Table S3. PCC sequences used in this study. 19

Table S4. Genetic element sequences used in this study. 41

Table S5. Kinetic parameters of PCC complexes and 6-dEB titers of the corresponding *E. coli* strains. . 41

Supplementary References 42

# Materials and Methods

## Materials and Laboratory Services

Chemicals were obtained from Sigma-Aldrich unless otherwise noted. Primers for PCR and small DNA fragments were ordered from Integrated DNA technologies (IDT). Sequencing was performed at Sequetech, Mountain View, CA. Restriction enzymes were obtained from New England Biolabs (NEB), T5 exonuclease from Epicentre, Phusion DNA polymerase from NEB, and Taq DNA ligase from NEB. Phusion^®^ Hot Start II High-Fidelity DNA Polymerase (Finnzymes) was used for all PCR reactions. DNA fragments for constructing the *propionyl-CoA carboxylase* genes were a gift from Gen9 (<http://www.gen9bio.com/>). Unless otherwise specified, propagation of all *E. coli* strains harboring plasmids was performed in Luria-Bertani (LB) media containing the appropriate combination of carbenicillin (carb, 50 mg/ml), kanamycin (kan, 50 mg/ml), or a combination thereof.

## Bacterial Strains

For protein expression experiments, two *E. coli* strains were used: T7 Express (NEB) (genotype: fhuA2 lacZ::T7 gene1 [lon] ompT gal sulA11 R(mcr-73::miniTn10--Tet^S^)2 [dcm] R(zgb-210::Tn10--Tet^S^) endA1 Δ(mcrC-mrr)114::IS10) and BL21(DE3) (genotype: *F^-^ ompT hsdSB (r_B_^-^, m_B_^-^) gal dcm λ(DE3).* *E. coli* DH5α was used for general cloning (genotype: *fhuA2*Δ*(argF-lacZ)U169 phoA glnV44* Φ*80* Δ*(lacZ)M15 gyrA96 recA1 relA1 endA1 thi-1 hsdR17*). *E. coli* BAP1 was used for 6-dEB production (*BL21(DE3); ΔprpRBCD: T7prom-sfp-T7prom-prpE*)^1^.

## Selection of pcc genes for heterologous expression

A search of the NCBI PubMed database for the terms “propionyl-CoA carboxylase” or “propionyl CoA carboxylase” resulted in 607 abstracts, 153 of which contained a species name. PCC complexes from nine different species were selected from this list based on experimentally verified PCC activity, as opposed to ACC activity and/or hypothetical proteins. An additional 20 homologous PCC complexes from different species did not have experimentally verified PCC activity, but shared high sequence similarity with the above nine. These 29 protein sequences were identified in NCBI and entered the DNA synthesis process. Of these, we were able to assemble 13 full-length, correct alpha and beta subunit genes. These 13 genes originated from eukaryotes, alpha-proteobacteria, delta-proteobacteria, and actinobacteria.

## Construction of PCC plasmids

All plasmids are described in Table S1. The *pcc* gene homologs were provided as ~800bp double stranded synthetic DNA fragments by Gen9 (referred as Gen9 fragments below). All Gen9 fragments were codon optimized for *E. coli* using DNAWorks software^2^, with class II codon frequencies of highly expressed genes (Table S3). The Gen9 fragments were designed to have 42 bp overlap with each other to facilitate assembly. The sequences of all genetic elements upstream and downstream of each *pcc* gene are listed in Table S4.

Assembly of PCC plasmids (pGV404s for α-subunits and pGV405s for β-subunits) were performed via Gibson assembly^3^. Three Gen9 fragments were required to assemble one *pccA* gene, encoding the α-subunit (~2 kB on average) and two fragments were required to assemble one *pccB* gene, encoding the β-subunit (~1.5 kB on average). A pET21c-derived vector backbone was obtained by PCR amplification. 30 ng of each Gen9 fragment were added to 20 ng vector in 10x molar ratio in the assembly mixture. The 20 μl reaction was incubated for 1 hour at 50°C.

The synthetic Gen9 fragments were not clonally sequence-verified prior to the assemblies (error rate ~1/1500). To enrich for correctly assembled genes, a neomycin phosphotransferase selectable marker (*kanR*) was fused downstream of each *pcc* gene. 5 μl of assembly mixture were directly transformed into T7 Express or BL21(DE3) *E. coli* Z-competent cells (Zymoresearch) following the recommended protocol for transformation into T7 Express competent cells (NEB) and plated on carbenicillin-containing media. Colonies were replica plated onto media containing kanamycin (25 mg/ml) and isopropyl β-D-1-thiogalactopyranoside (IPTG) (1mM). Clones that grew in the presence of kanamycin putatively expressed the *kanR* gene fused to the *pcc* gene, indicating that no deleterious mutations led to early stop codons upstream of the selective fusion. Additionally, growth on kanamycin may select for PCC subunits that are properly folded, as misfolding could lead to aggregation and loss of activity of the *kanR* fusion. Clones were sequenced, and the C-terminal in-frame fusion approach reduced the error rate from 1/1500 to 1/4000.

Individual sequence-verified α and β-subunits were subsequently joined onto one vector (pGV407 plasmid series). *pccA* and pET21c-*pccB* fragments were PCR-amplified from pGV404s and pGV405s, respectively. All PCR-amplified fragments were treated with DpnI and purified using QIAquick Spin Purification Kit (Qiagen). ~50 ng of *pccA* and ~150 ng of pET21c-*pccB* fragments were joined by Gibson assembly in a molar ratio 10x. 5 μl of assembly mixture were transformed in DH5α Z-competent *E. coli* cells and plated on selective medium (LB + carbenicillin). Correctly assembled clones were verified by colony PCR and sequencing. 1-2 mismatches that were not nonsense mutations were allowed.

## Construction of MH109

In order to generate strains of *E. coli* capable of polyketide production, MH109, a single plasmid with all *debs* genes, was constructed. *debs1 w*as amplified from pBP144^1^, harboring the *S. coelicolor* *pcc* genes and *debs1*. *debs2* and *debs3* were both PCR-amplified from pBP130^1^. *E. coli* and *S. cerevisiae* origins of replication and selectable markers were PCR-amplified from pET28a and pRS415, respectively. MH109 was assembled via a 7-fragment yeast homologous recombination.

## Construction of second-generation PCC plasmids

pGV204 was constructed by PCR-amplification of α and β-subunits from pBP144. PCC subunits were subsequently joined with pET21c into pGV204 using an optimized Gibson assembly protocol: for GC-rich sequences, 5% dimethyl sulfoxide (DMSO) was added to the master mix, and the assembly reaction was incubated at 50°C for 15 minutes instead of 60 minutes. To construct pGV107, α and β-subunits were PCR-amplified from pBP144 and assembled, via the optimized Gibson assembly protocol as described above, with four short oligos (IDT) harboring (a) the optimized RBS and (b) the initial 20 codons optimized to match *E. coli* codon usage. pGV204-1 was constructed by PCR-amplification of α and β-subunits from pGV204 by excluding the C-terminal His_6_ tag on α-subunit. α and β-subunits were joined using the optimized Gibson assembly protocol as described above. pGV501 was constructed by PCR-amplifying α and β-subunits from pGV407-*S. coelicolor* and pGV407-*M. fulvus*, respectively, by excluding the β-subunit C-terminal Flag tag and the α-subunit N-terminal His_6_ tag. α and β-subunits were joined via Gibson assembly. Genscript constructed pGV502 from pGV407-*M. fulvus* plasmid by removing the β-subunit C-terminal Flag tag and the α-subunit N-terminal His_6_ tag. pGV503 and pGV504 were constructed by PCR-amplifying β-subunit and α-subunit, respectively, from pGV407-*S. coelicolor,* and assembled via standard Gibson assembly.

## Quantification of 6-dEB production using a liquid chromatography/mass spectrometry (LC/MS) assay

Plasmids harboring *pcc* genes (pGV407 series) were co-transformed with MH109 into *E. coli* BAP1 by electroporation^4^ and the cells were plated on LB agar containing both kanamycin and carbenicillin. A single colony was picked from the transformed cells and used to inoculate a seed culture (5 ml LB media containing the appropriate antibiotics in a 15 ml Falcon tube). 53 ml of LB medium into 250 μl flasks with the appropriate antibiotics were inoculated with 600 μl of the seed culture. Cultures were incubated at 37°C at 250 rpm.

When OD_600_ reached 0.4 - 0.5, the cultures were induced with 700 μl of 0.1 mM IPTG, and sodium propionate was added at a final concentration of 2.5 g/l. After growth for 48 hours at 18.5°C, culture supernatants were clarified by centrifugation at 3600 rpm for 5 min and stored at -20°C prior to LC/MS analysis. 100 μl of each supernatant was diluted with 900 μl of acetonitrile in a microtiter plate. These diluted samples were analyzed by injection of 1 μl of sample into the LC/MS system using a Zorbax RRHD Eclipse C18 column (50 mm x 2.1 mm x 1.8 μm). Mobile phases were 0.1% v/v formic acid in either water (A) or acetonitrile (B). Elution was isocratic at 5% B for 0.2 min, and increased to 79.25% B in a linear gradient from 0.2 min to 3.5 min. The column was subsequently washed with 95% B for 0.5 min and 5% B for 1 min. The mass spectrometer was an Agilent 6545 qTOF fitted with a dual AJS electrospray ion source in positive ion mode. 6-dEB eluted at 2.8 minutes and was detected as the sodium adduct (C21H38O6 + Na)+) at an m/z of 409.2561.
Authentic 6-dEB was prepared as described previously^5^. Extracted ion chromatograms and mass spectrum for the authentic standard are shown in Figure S6. During LC/MS runs for analyzing 6-dEB titers, a full standard curve was prepared by diluting aliquots of authentic 6-dEB into LB medium (Figure S7). Peak areas from standards were regressed against known concentrations (intercepts were forced to 0) to calculate response factors. The relative standard error of the response factor was less than 10% in all cases, despite substantial (~30%) variation in the response factor from run to run. Three replicates were obtained from three separate fermentations. The fermentation cultures were started from the same *E. coli* clones.

## Protein Expression of PCC subunits

40 ml of the liquid cultures used for the 6-dEB quantification were centrifuged at 3600 rpm for 20 minutes to pellet the cells and were stored at -20°C prior to protein purification. α and β-subunits were purified using Qiagen Ni_NTA spin protocol under denaturing conditions. Cell pellets were lysed by addition of 1.4 ml lysis buffer (7 M urea; 0.1 M NaH_2_PO_4_; 0.01 M Tris·Cl; pH 8.0) and 3 Units/ml culture volume Benzonase Nuclease (Sigma-Aldrich), and they were incubated with agitation at room temperature for 30 minutes. After centrifugation at 12,000 g for 30 minutes, the clear cell lysates were normalized to a final concentration of 1 mg/ml using the Bradford protein assay^6^. 600 μl of the normalized cell lysate was loaded onto pre-equilibrated Ni-NTA spin column. The resin was washed with 4 column volumes of wash buffer (0.1 M NaH_2_PO_4_; 0.01 M Tris·Cl; pH 6.3). Bound proteins were eluted twice in 100 μl elution buffer (0.1 M NaH_2_PO_4_; 0.01 M Tris·Cl; pH 4.5). 18 μl samples were visualized on Novex® NuPAGE® 4-12% Bis-Tris Precast Gel (Life Technologies) for 2 hours at 120 V. Semi-quantitative densitometry analysis of protein level differences was performed using ImageJ software version 1.50i (Image Processing and Analysis in Java, https://imagej.nih.gov/ij/). The analysis provides the ratio of densitometry measured band densities between wild type and optimized α-subunit of *S. coelicolor*.

## Western blotting of α-subunits

To measure biotinylation of the α-subunits from pGV407s plasmids, 10 μl of normalized clear cell lysates were visualized on Biorad 4–20% Mini-PROTEAN® TGX™ Gel for 30 min at 200 V, as pGV407-*C. glutamicum* PCC and pGV407- *P. acnes* PCC were diluted 5x and pGV407-*Paracoccus sp.* PCC, and pGV407-*S. coelicolor* PCC were diluted 10x for better visualization. Proteins were electroblotted onto nitrocellulose membrane in Transfer buffer (20 mM Tris, pH 8, 150mM glycine, 20% (v/v) methanol) at 80 V for 1 hour. The membrane was washed in Blocking buffer (1x PBS, pH 7.5, 0.1% (v/v) Tween20, 5% (w/v) Bovine Serum Albumin) overnight at 4°C. The membrane was rinsed with Wash buffer (1x PBS, pH 7.5, 0.1% (v/v) Tween20) and incubated with a 1/10,000 dilution of Streptavidin-HRP conjugate for 1 hour at room temperature. After rinsing the membrane with wash buffer, ECL-2 Western Blot Solution (Pierce/Thermo Fisher) was added to the membrane and incubated for 30 sec. Biotinylated proteins were visualized using a Typhoon 8600 imager.

The same protocol was used to verify biotinylation of α-subunits from pGV204 and pGV107. Prior to Western blotting, cleared cell lysates from pGV501-504 were normalized to 1 mg/ml, and cleared cell lysates from pGV204 and pGV107 were diluted to 0.1 mg/ml and 0.2 mg/ml, respectively.

# Supplementary Figures

## Figure S1. Protein expression of α and β-subunits of the wild-type and computationally-optimized S. coelicolor PCC constructs (construct #1 and #2 from Figure 1).

(A) Coomassie –stained denaturing protein gel of the two constructs in the soluble fraction. Samples diluted 100x. (B) Streptavidin Western blot of the α-subunits of the computationally-optimized *S. coelicolor* PCC construct (#2 from Figure 1).


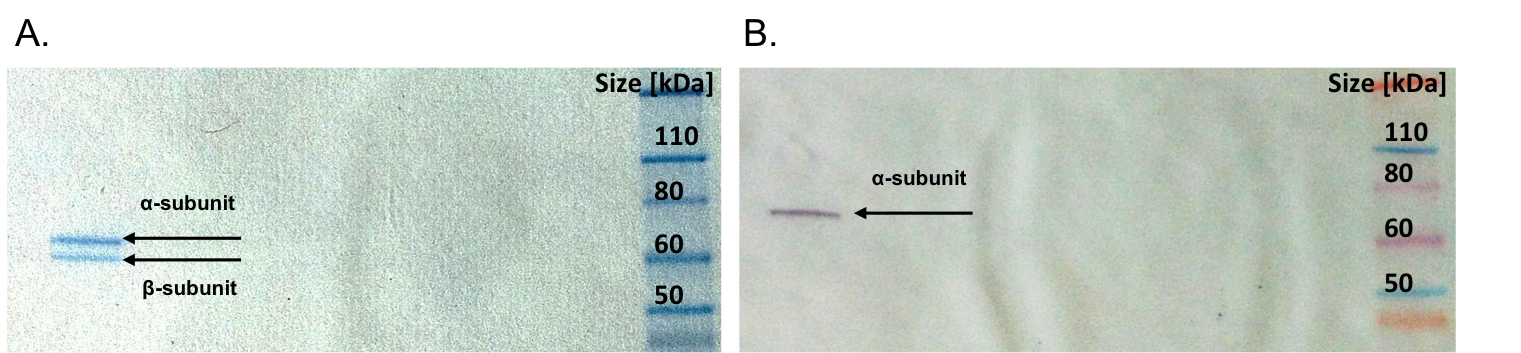


## Figure S2. (A) Phylogenetic tree of 13 PCC α-subunits and E. coli ACC BC subunit and (B) phylogenetic tree of 13 PCC β-subunits and E. coli ACC CT subunit.

Alignments were generated using Clustal Omega^7^. The evolutionary history was inferred by using the maximum likelihood-based RAxML^8^ with the PROTCATJTT^9^. Phylogenetic unrooted tree was visualized in FigTree. (<http://tree.bio.ed.ac.uk/software/figtree/>). Color legend: Eykaryotes: magenta, Actinobacteria: blue, Alphaproteobacteria: red, Deltaproteobacteria: green, *E. coli* ACC: black.

A.

B.

## Figure S3. Protein expression of engineered PCC α and β-subunits homologs.

(A) Coomassie –stained denaturing protein gel of Ni-NTA-purified (upper) and clear cell lysates (bottom panel) PCC subunits in the soluble fraction. α and β-subunits labels are shown on the predicted molecular weight. The 13 PCCs studied in this work are labeled. (B) Coomassie –stained denaturing protein gel of clear cell lysates (left) and streptavidin Western blot of the α-subunits of the engineered PCC homologs from clear cell lysates (right). (*) pGV407-*C. glutamicum* PCC and pGV407- *P. acnes* PCC were diluted 5x. (**) pGV407-*Paracoccus sp.* PCC, and pGV407-*S. coelicolor* PCC were diluted 10x to avoid overloading of the Western blotting gel.


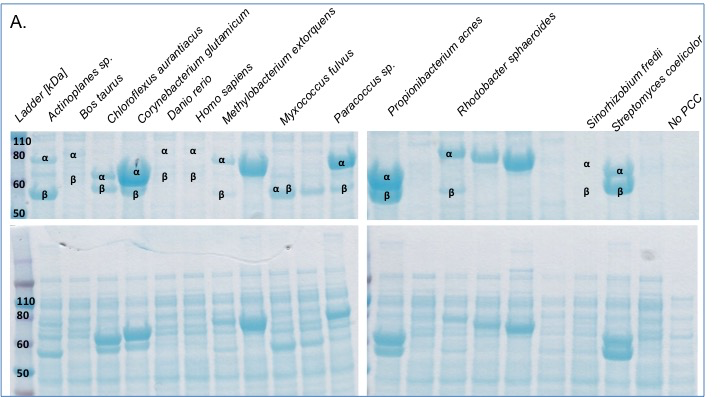


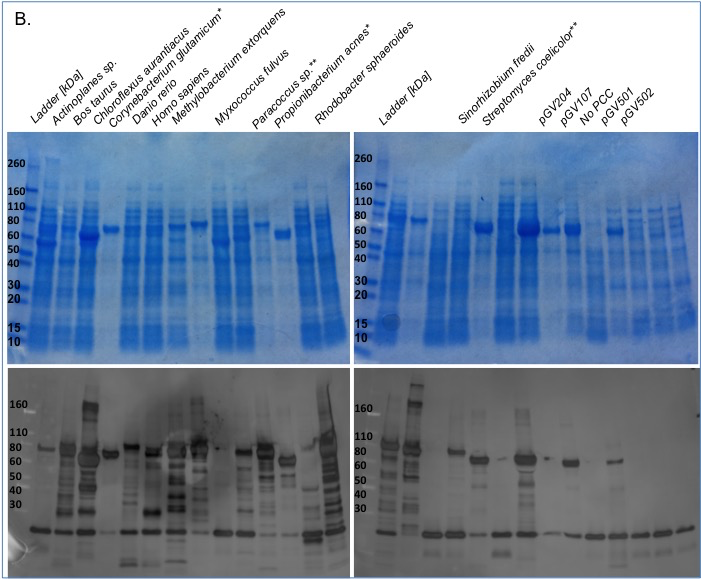


##

## Figure S4. Scheme of wild-type S. coelicolor PCC.


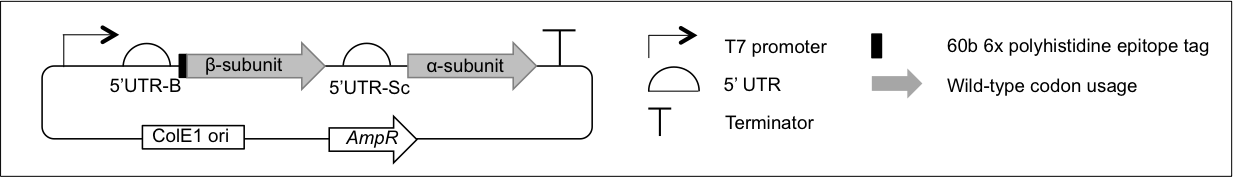


## Figure S5. Graphic display of the PCC genome region of M. fulvus.


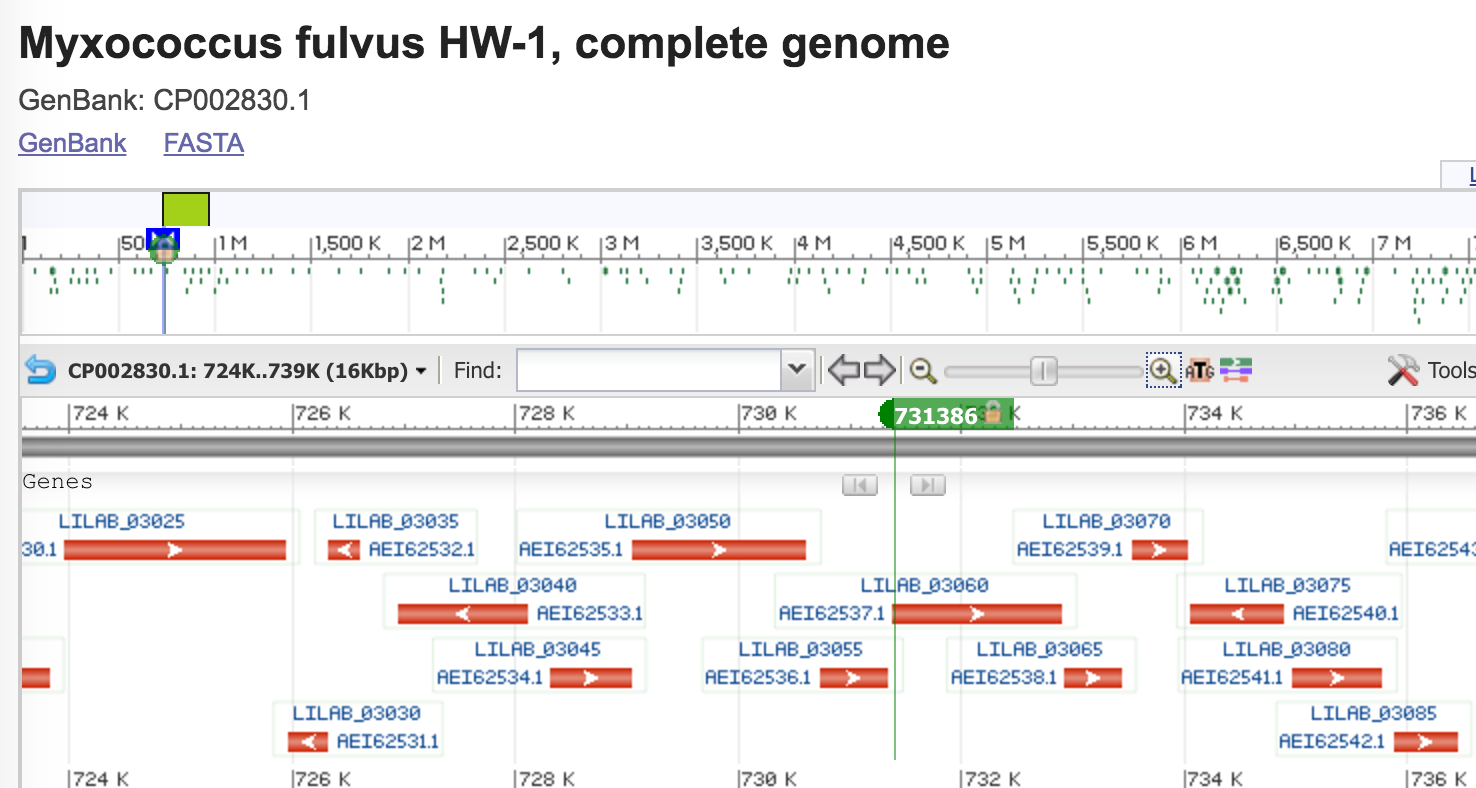


PCC beta

Hypothetical protein

PCC alpha

Biotin carboxyl carrier protein

## Figure S6. LC/MS analysis of authentic standard 6-dEB.

(A) Extracted ion chromatograms (obtained by extraction of the [M+Na]+ species) for authentic standard 6-dEB. (B). Full (left) and zoomed (right) mass spectra for authentic standard of 6-dEB.


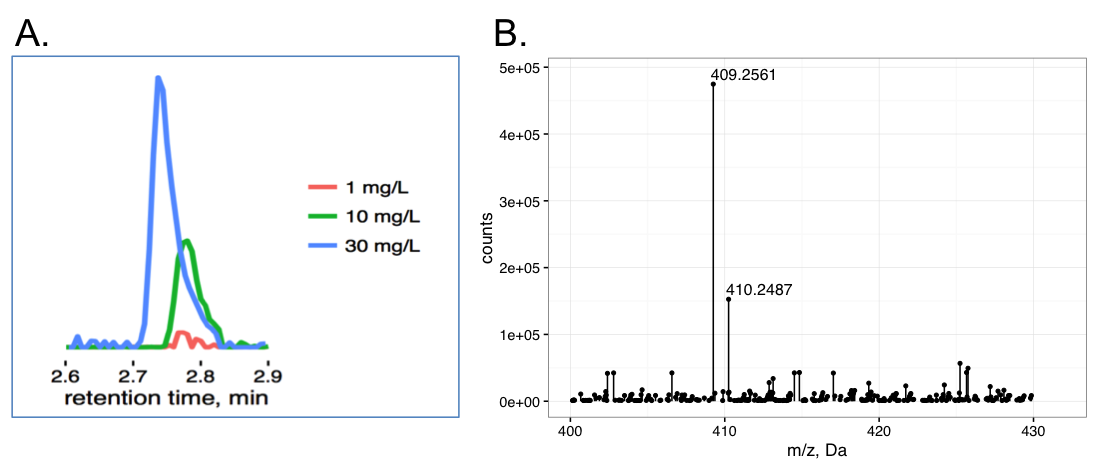


## Figure S7. LC/MS standard curves of an authentic standard of 6-dEB run on both days LC/MS data was collected.


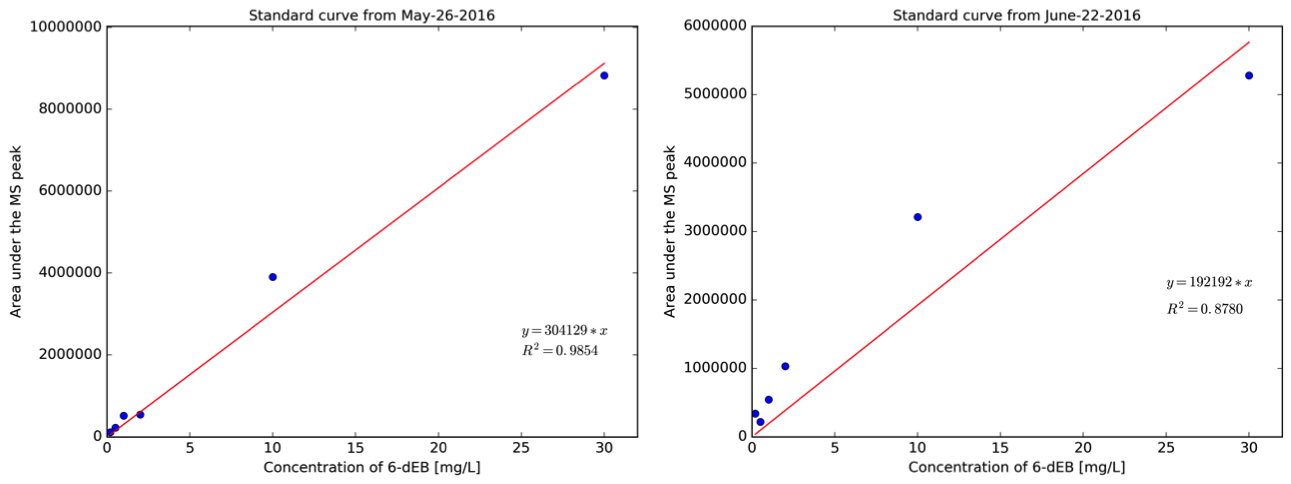


# Supplementary Tables

## Table S1. Plasmids used in this study.

kan: kanamycin, carb: carbenicillin; T7prom: T7 promoter, 5'UTR: 5' untranslated region; *pccB*: propionyl-CoA carboxlase β-subunit; *accA1*: acyl-CoA carboxylase α-subunit; *pccA*: propionyl-CoA carboxylase α-subunit; *debs*: 6-deoxyerythronolide-B synthase, 5'UTR-Sc - wild-type 5'UTR of S. coelicolor; 5'UTR-Salis: optimized by Salis tool 5'UTR; N-his: N-terminal 6x polyhistidine tag; C-his: C-terminal 6x histidine tag; N-Flag: N-terminal Flag tag; C-Flag: C-terminal Flag tag; *kanR*: neomycin-resistance gene.

| **Name** | **Backbone** | **Resistance** | **Description** | **Reference** |
| --- | --- | --- | --- | --- |
| pBP144 | pET28a | kan | T7prom-5'UTR-B-*pccB*-UTR-Sc-*accA1*-T7prom-5'UTR1-*debs1*-T7term | Pfeifer 2001^1^ |
| pBP130 | pET21c | carb | T7prom-5'UTR2-*debs2-5'UTR3*-*debs3*-T7term | Pfeifer 2001^1^ |
| pMH109 | pET28a | kan | T7prom-5’UTR1-*debs1*-T7term*-*T7prom-UTR2-*debs2-5’UTR3*-*debs3*-T7term | this work |
| pGV204 | pET21c | carb | T7prom-5'UTR-B-N-His6-*pccB*-5'UTR-Sc-*accA1*-C-His-T7term | this work |
| pGV204-1 | pET21c | carb | T7prom-5'UTR-B-N-His-*pccB*-5'UTR-Sc-*accA1*-T7term | this work |
| pGV107 | pET21c | carb | T7prom-5'UTR-B-N-His-*pccB*-5'UTR-Salis-*accA1*-C-His-T7term | this work |
| pGV404s | pET21c | kan, carb | T7prom-5'UTR-A-N-Flag-*pccA*-C-His-*kanR*-T7term (*pccA* genes from 13 species) | this work |
| pGV405s | pET21c | kan, carb | T7prom-5'UTR-B-N-His-*pccB*-C-Flag*-kanR*-T7term (*pccB* genes from 13 species) | this work |
| pGV407s | pET21c | carb | T7prom-5'UTR-B-N-His*-pccB*-C-Flag-5'UTR-A-N-Flag*-pccA*-C-His-T7term *(pcc* genes from 13 species) | this work |
| pGV501 | pET21c | carb | T7prom-5'UTR-B-N-His-*pccB*-5'UTR-A-*pccA*-C-His-T7term (*pcc* genes from *S. coelicolor*) | this work |
| pGV502 | pET21c | carb | T7prom-5'UTR-B-N-His-*pccB*-5'UTR-A-*pccA*-C-His-T7term (*pcc* genes from *M. fulvus*) | this work |
| pGV503 | pET21c | carb | T7prom-5'UTR-B-N-His-*pccB*-T7term *(pccB* gene from *S. coelicolor*) | this work |
| pGV504 | pET21c | carb | T7prom-5'UTR-A-*pccA*-C-His-T7term (*pccA* gene from *S. coelicolor*) | this work |

## Table S2. List of PCC enzyme homologs used in study.

| **Organism** | **PCC subunit** | **Accession number** | **Length [amino acids]** |
| --- | --- | --- | --- |
| *Actinoplanes sp.* | α-subunit | AEV88984.1 | 667 |
| *Bos taurus* | α-subunit | NP_001076978.1 | 741 |
| *Chloroflexus aurantiacus* | α-subunit | ABY34606.1 | 596 |
| *Corynebacterium glutamicum* | α-subunit | BAB98093.1 | 591 |
| *Danio rerio* | α-subunit | NP_001002746.1 | 709 |
| *Homo sapiens* | α-subunit | NP_000273.2 | 728 |
| *Methylobacterium extorquens* | α-subunit | ACS40950.1 | 667 |
| *Myxococcus fulvus* | α-subunit | AEI62537.1 | 505 |
| *Paracoccus sp.* | α-subunit | WP_010396162.1 | 685 |
| *Propionibacterium acnes* | α-subunit | AEE72932.1 | 589 |
| *Rhodobacter sphaeroides* | α-subunit | ABA78345.1 | 695 |
| *Sinorhizobium fredii* | α-subunit | CCE99604.1 | 678 |
| *Streptomyces coelicolor* | α-subunit | WP_011029950.1 | 590 |
| *Actinoplanes sp.* | β-subunit | AEV84051.1 | 524 |
| *Bos taurus* | β-subunit | DAA33089.1 | 539 |
| *Chloroflexus aurantiacus* | β-subunit | ABY35246.1 | 535 |
| *Corynebacterium glutamicum* | β-subunit | YP_227111.1 | 516 |
| *Danio rerio* | β-subunit | NP_998090.1 | 557 |
| *Homo sapiens* | β-subunit | EAW79118.1 | 559 |
| *Methylobacterium extorquens* | β-subunit | ACS38134.1 | 510 |
| *Myxococcus fulvus* | β-subunit | AEI62535.1 | 520 |
| *Paracoccus sp.* | β-subunit | ZP_08665243.1 | 534 |
| *Propionibacterium acnes* | β-subunit | AEE72921.1 | 524 |
| *Rhodobacter sphaeroides* | β-subunit | ABA78341.1 | 510 |
| *Sinorhizobium fredii* | β-subunit | CCE99606.1 | 510 |
| *Streptomyces coelicolor* | β-subunit | 1XNY_B | 530 |

##

## Table S3. PCC sequences used in this study.

| **Organism** | **PCC subunit** | **ORF DNA Sequence** |
| --- | --- | --- |
| *Actinoplanes sp.* | α-subunit | ATGATTCGTCGTCTCCTCGTAGCCGACCGTGGCGAGATCGCTCGTCGTGTTTTCGCTACC TGTCGCCTCGTGGGCATCGAAACCGTTGCAGTCTACTCTGATGTCGACTCCGATGCGCCG CATGTGAATGAGGCTGATTACGCTGTACACCTGGAAGGCTCTACCCCAGCGGCGACGTAT CTCGAGCAACACGCACTCATTGCCGCAGCGAAGCGTGCGGGCGCTAATGCAGTACATCCA GGTAACGGCATGCTGGCGGAAGACCCAGAATTCGCAGCCGCTGTTAGCGATGCGGGTATG ATCTGGGTTGGCCCACCGGCAGCAACTCTGCGCACCCTGCACCGTAAAACCGAAACCAAG AAACTGGTTGCCGAAGCTAAAGTACCGGTTCTCCCGTCTTTCACCGACCCGGAGACTGTT CCGGGTTTCCCTGTACTCATTAAGCCAGACACGGGTCTGGGTGGCGCTGGTATGCGTGTG GTACGTGACGCTGCTACGCTGGCCGAGGCCGTCGCAAGCACGCGTCGCGAGGTAGATGGC GATGTGTTTTGCGAGCCATACGTTCCGAATGTGCGTCACATCGAGGTGCCAATTCTCGCA GATGCCATGGGCGCTGTAGTACCGTTTGGTGAACGTGAATGCTCCGTTCAGCGTCGTTAC CAGAAAATTGTTGAAGAAACGCCGTCTCCAGCCGTAGACGCAGCTCTCCGCGAAGACCTG TGCCGTGCCGCAATCGTTGCGGTACGCGCTCTCGGCTTCGTTGGTGCCGGCGCGGTAGAA TTTCTGCTGGACGCGGACGGCGACTTTTGGTTCCTCGAACTGACCCCTACGCTCCAGACT GAACACGCGGTAACCGAGTGTACCTCTGGTTATGACCTGGTTCGCCTCCAGCTGCTGGTG GCAGAGGGCGGTTCCCTGCCGATGCCAGGCCCTCCACCTATCCGTGGTCACGCCATTGAG GTTCGCATTTGCGCGGAGGATCCGGCCTACGCGTGGCTCCCGAGCAGCGGCACTCTCCAC CGTTTCGCGGTACCTGACGTAGCGGGTGCGTTCCGTCCTCTCCCACAGCCGGGCCTGCGC CTCGATTCCGGTGTTGCAGACGGCAGCGTTACCGGTCAACATCATGAACCTATGCTGGCT AAGCTCGTGGCCTGGGCGCCTTCTCGTCAAGAGGCTGCCCGCATGCTCGCCAGCGCTCTG GCGCGTGCGCAACTGCATGGTGTAGTTACCAACCGTGACCTGCTCGTTCGTACCCTCCGT CATCACTCTTTTCGCGCGGGTGAGATTGACACCGGTTTCCTGGACCGCCATCCGGAGGTC TTTGCCCCGCTCCTCAGCTCTGTTGATGCAGTCCGTCTGTCCTGCCTCGCTTCTGCTCTC GCCGGTGCGGCTGACCGCCGTGCATCCGCTCCGGTCCTGGGTACTCTGCCTTCTGGCTGG CGTAATGTCCCATCTGGCTCTCAGACTGCGGTATACGATGGTCCAACCGGTCCGGTCGAA GTCGGTTATCGTATGAACCGTCATGGCGAACTCGCAGGTTGGTGGGTCCGTCCAGTTGAT CCGGAAGAGCTGGATCTGGCAGGCATTGGTCAAGCGCCTGTTGACGAACACCCGCCGATC GTGGTAGTTTCCGCATCTCGTGACCGTGTCGTTCTCAACGTTCAGGGTATCCGCCTGGCT GTTCTCGTTCATCGTGTGGGTGACGTCTCTTACGTGGACAGCCCGGAAGGTTCTGTTACC CTGCGTGAAATCTCTCGTTTCCCACTCCCAGCCCCAGAAGCTGGTGAATCTTCTCTGATC GCCCCACTGCCTGGCGCAGTACGCCGCGTTCTGGTTGTTCCTGGTCAGCGCGTCCGTGCA GGTGAACTCCTGCTCACGCTCGAAGCGATGAAGCTCGAACATCCGGTTCACGCCCCAAGC GCAGGTGTTGTAGCAAGCCTCCCTGTTCACCCTGGTGCTGAGGTTGCCACTGGTGAGCTC CTCGCAGTCCTCGATCCAGAA |
| *Bos taurus* | α-subunit | ATGGCTGGTTTTTGGACTGGTGCGGCAGCACTGGCGACCGCAGGCCGTCGTGGTCGCCGT TGGCCTAAACTGCTGATGCGTTCTGTCGCACTGTGGACCGTAAAACACGTTCCACATTAC AGCCGCCGTTATCTCGTAGTTTCTCGTACGCTGTGCTCTGCGGGTTACGATTCCAACGAG AAGACCTTCGACAAAATCCTCATCGCGAACCGCGGCGAAATTGCGTGCCGTGTGATTAAA ACCTGCAAGAAAATGGGCATCAAGACCGTTGCCATCCACTCTGACGTGGACGCATCTTCC GTTCACGTTAAAATGGCGGACGAGGCTGTGTGCGTTGGTCCGGCACCGACGTCCAAATCC TACCTCAACATGAACGCCATCATGGACGCCGTTCGCTCTACTCGTGCACAAGCAGTGCAC CCGGGCTACGGCTTTCTGTCTGAGAACAAAGAGTTTGCCAAACGCCTCGCAGCTGAAGAT GTGATCTTTATCGGTCCTGACACGCACGCGATCCAAGCCATGGGTGACAAGATCGAATCT AAGCTGCTGGCCAAGAAAGCAAAAGTCAACACGATCCCTGGCTTTGACGGTGTCGTTAAG GATGCTGACGAAGCGGTACGCATCGCCCGTGAGATCGGCTACCCTGTTATGATCAAGGCA TCTGCTGGTGGCGGTGGTAAAGGCATGCGTATCGCCTGGGACGACGAAGAAACCCGTGAC GGTTTCCGTTTCTCTTCTCAGGAAGCCGCATCCTCTTTTGGCGACGACCGTCTCCTGATC GAAAAATTCATCGACAACCCGCGTCACATCGAAATTCAAGTTCTCGGCGACAAACACGGT AATGCGCTGTGGCTCAACGAGCGTGAGTGCTCTATTCAGCGTCGTAATCAGAAGGTTGTG GAGGAAGCGCCGTCTATCTTTCTGGATAGCGAAACTCGCCGTGCAATGGGCGAACAAGCG GTGGCACTCGCCAAGGCGGTTAATTACTCTTCTGCCGGTACGGTTGAGTTCCTCGTCGAC AGCAAGAAGAACTTCTACTTCCTCGAGATGAATACGCGCCTCCAGGTCGAACACCCAGTA ACTGAATGCATCACCGGTCTGGATCTGGTACAGGAAATGATCCGTGTGGCCAAAGGTTAC CCTCTGCGTCATAAGCAGGCGGACATCCCGATCAACGGCTGGGCAGTTGAATGCCGCGTT TACGCGGAGGACCCGTATAAATCTTTCGGCCTCCCATCTGTTGGCCGTCTGTCTCAGTAT GAAGAACCGATTCACCTGCCGGGTGTACGTGTCGATTCTGGTATCCAGCCAGGCTCTGAC ATTAGCATCTACTACGACCCGATGATCTCCAAGCTCATCACGTACGGCTCCGATCGTACC GAAGCGCTGAAGCGTATGGAGGACGCGCTGGACTCTTACGTTATCCGTGGTGTGACCCAC AACATTGCCCTCCTGCGTGAAGTTATCATCAACTCTCGCTTCATTGAGGGCGACATTAAC ACCAAGTTCCTGTCCGACGTTTATCCGGACGGCTTCAAAGGTCATCCGCTCACCGAAACC GAGCGCAACCAATTCCTCGCGGTTGCTAGCTCTCTGTTTGTGGCGAGCCAGCTCCGTGCG CAACACTTCCAGGATCACGAGTCTTCCCGCGTTCCAATTGTGAAGCCTCAAGTTGCCTCT TGGGAACTGAGCGTCAAGCTGCACGATGAGATCCACACCGTTGTTGTATCTTCCTCTGGC CCGACTTTTTCTCACGAGCCGCAAAAACTCCTGCACAGCTACATGGTAGAGGTCGACGGT AGCAAGCTGAATGTAACTAGCACGTGGAATCTGGCGTCTCCGCTGCTCTCCGTGAATGTT GACGGCACCCAACGTACCATTCAATGCCTCTCTCGTGAAGCAGGTGGCAACATGTCCATC CAGTTTCTGGGCACCGTTTACAAAGTTCGTATCCTGACCAAGCTGGCGGCCGAGCTCAAC AAGTTCATGCTCGAGAAAGCGGCGGAAGACACCTCTTCTATCCTGCGCTCCCCTATGCCT GGTGTTGTAGTTGCGATCTCTGTCAAACCGGGTGACCTGGTTGCTGAGGGCCAGGAGATC TGTGTCATCGAGGCCATGAAAATGCAGAACAGCATGACCGCCGGTAAAACCGGCAAGGTT AAGAGCGTACACTGCAAGGCTGGTGATACCGTTGGTGAAGGCGATCTGCTCGTAGAGCTG GAA |
| *Chloroflexus aurantiacus* | α-subunit | ATGTTCCGTACGATCCTGGTTGCAAACCGTGGCGAGATCGCGCTCCGTGTTATGCGCGCG TGCCGCGAGCTGGGCCTCCGTTGTGTAGCGGTATACTCTGAGGCCGATCGTGATGCGCCG CACGTTGCTTACGCGGATGATGCTTTCCTGATCGGTCCACCGAGCCCAGCCGAGTCCTAC CTGAACATCGACGCCATCATTCGTGCTGCGAAAGCCACGGGTGCTGAAGCCATTCACCCG GGCTACGGTTTCCTCGCGGAAAATGCCTCTTTTGTGCGCGCTGTGACGGCTGCAGGCCTC ATCTTCATTGGCCCACCAGCGGAGGCGATGGAGCGTATGGGCGGTAAGACCGCGGCGCGT CGCGAGGCCACGGCAGCTGGTGTTCCTGTTGTTCCGGGTGTTCTGGAACCAGTTACCGAT GCTGCCGAGGTGCGTCGCCTGGGTAAGGAATTTGGTTACCCGATCGCAATCAAAGCGGTG GGTGGCGGTGGTGGTCGTGGTCTGCGCGTTGTTCGTTCCCCAGAAGAAGTTGACGAAGCA TTCGCTGCGGCTCGTCGTGAAGCCGAAGTGGCGTTTAAGAATGGCGAGCTCTACGTCGAA AAGTACCTGGACGACCCACGCCATATTGAAATTCAGGTGCTGGCGGATCGTTATGGCAAT GCGGTAGCCCTGGGCGAACGTGACTGCTCTGTTCAGCGTCGTCACCAAAAACTCATCGAG GAGTGCCCGTCCCCGGCTCTCACTCCTGAACTGCGTGCGGAAATGGGCGCAGCGGCGGTG CGCCTCGCCAAGGCGGTTGGCTACGTTTCTGCTGGTACCCTGGAGTTCCTCTTCCAGGAC GGTCGTTACTACTTCCTCGAAATGAATACCCGCATCCAGGTCGAACACACGGTTACGGAG ATGGTTTACGGTATTGACCTCGTGGCAGCACAAATTCGTATCGCGCAGGGTGAGAAACTG TGGTTCAAACAAGAGGACGTTGTACCGCGTGGTCACGCAATCGAGTGCCGCATCAATGCA GAAGATCCGCTCCACAACTTTCGTCCGGCCCTCGGTACTATCGGTGAGTACCATGAACCG GTCGGTTTTGGCGTGCGTGTTGACTCTGGTGTCCGTGCGTACTACACCGTGCCATCTCAC TACGACTCTCTCCTGGCGAAGCTGATCACCTGGGGTTCTGATCGTCAGGAAGCTATTGCG CGTATGCGTCGTGCGCTGGCCGAGTATCGCATCGAAGGCGTTACCACCATTATCCCGTTC CACCAGGCCGCTCTGGAGCATCCGGTGTTCACGGCGGGTGCCGCTACTGTAAACTTCATT CCGCGTCATCCGGAGCTGTTCTCTCGTGCAGCGGAACTGACGCCACCGACTGCGGCGTCC GCAGGCGCGGAGCCTGCGCCGGAGCCACGTCGTTTTACTATTGAAGTGAACGGTCGCCGC TTCGGCGTTGCAGTCTTTGGCGACGGTATGAACGCGACCCCGGTTGCGTCCCCTTCTCGC TCTGCCCCTGCACGTCGCGCTGCCCCGAAAAAGACCACCCTGGCTGCACCGGTAGACGCT GTTATTTCTCCGATCCAAGGTCGTGTCGTGGCCGTTCGCGTAGCGCATGGTCAACAAGTT GAAGCGGGTCAAGTGCTGTTTATCGTCGAGGCAATGAAAATGGAGAATGAGATTACCGCG CCACATTCTGGCACGATTGCTGAGGTTCGTGTGGAAGTCGGCACTACCGTAGAGGCCGGT GCGATGCTCGCTACCTATCAGAATACCGCGAATAATACCAACGGTAAA |
| *Corynebacterium glutamicum* | α-subunit | ATGTCTGTTGAAACCCGTAAAATCACCAAAGTTCTGGTTGCGAACCGTGGTGAAATCGCC ATTCGTGTTTTCCGTGCTGCCCGCGACGAAGGCATCGGTAGCGTAGCGGTATACGCAGAG CCAGATGCTGACGCCCCGTTTGTGTCCTACGCCGATGAAGCGTTCGCCCTCGGTGGCCAA ACGTCTGCGGAGTCTTACCTCGTGATTGACAAGATCATCGACGCAGCTCGTAAAAGCGGT GCGGATGCTATCCACCCGGGCTACGGTTTCCTGGCGGAAAATGCAGATTTCGCGGAGGCG GTTATCAATGAGGGCCTGATCTGGATCGGTCCGTCTCCAGAGTCTATCCGTTCTCTGGGC GACAAAGTGACGGCACGTCACATCGCGGACACCGCAAAGGCGCCAATGGCTCCAGGCACG AAAGAGCCGGTCAAAGATGCGGCCGAGGTAGTTGCGTTTGCAGAAGAATTCGGTCTGCCG ATTGCTATCAAAGCGGCTTTTGGTGGCGGTGGTCGCGGTATGAAAGTTGCCTACAAAATG GAGGAAGTAGCAGACCTGTTCGAATCTGCGACGCGTGAAGCAACGGCGGCATTTGGCCGT GGCGAGTGCTTCGTTGAACGTTACCTGGACAAAGCGCGTCATGTTGAAGCGCAGGTTATC GCTGACAAACATGGCAACGTCGTAGTGGCGGGTACTCGCGACTGCAGCCTGCAGCGCCGT TTTCAAAAACTGGTAGAAGAGGCACCGGCACCATTCCTGACCGATGACCAGCGTGAGCGT CTGCACTCTAGCGCGAAAGCAATCTGTAAGGAGGCCGGCTACTATGGTGCGGGCACGGTT GAATACCTGGTTGGTTCTGATGGTCTGATCTCCTTCCTGGAAGTTAACACGCGCCTCCAG GTGGAGCACCCTGTTACGGAAGAAACCACCGGTATCGATCTGGTTCGTGAAATGTTTCGC ATCGCCGAAGGCCACGAACTCTCCATCAAGGAGGACCCTGCACCGCGTGGTCACGCCTTC GAGTTCCGTATTAACGGCGAAGACGCGGGTTCTAACTTCATGCCAGCTCCGGGCAAGATT ACCTCTTACCGTGAACCGCAAGGTCCGGGTGTTCGTATGGATTCTGGTGTCGTAGAAGGC TCCGAGATTAGCGGCCAATTCGATTCCATGCTCGCTAAGCTGATTGTTTGGGGTGACACC CGTGAGCAAGCGCTCCAGCGCTCTCGTCGTGCGCTCGCAGAATACGTTGTCGAAGGTATG CCGACCGTAATCCCGTTCCACCAGCATATCGTGGAGAACCCAGCTTTTGTCGGTAATGAT GAGGGTTTCGAGATTTATACTAAGTGGATCGAGGAGGTATGGGACAACCCGATCGCCCCA TACGTAGACGCCTCTGAACTGGATGAAGACGAAGACAAGACCCCAGCTCAGAAAGTGGTC GTCGAGATCAATGGCCGTCGTGTCGAGGTTGCACTGCCAGGCGACCTGGCGCTCGGCGGT ACTGCTGGCCCAAAAAAGAAAGCTAAAAAACGCCGTGCAGGCGGTGCCAAGGCAGGTGTA TCCGGTGATGCAGTTGCCGCTCCAATGCAGGGCACTGTGATCAAGGTTAATGTTGAGGAA GGTGCTGAGGTCAACGAGGGTGATACGGTAGTCGTACTCGAGGCCATGAAGATGGAAAAT CCAGTAAAGGCACACAAGTCTGGTACGGTCACTGGCCTGACGGTGGCAGCCGGTGAAGGT GTCAACAAGGGCGTTGTACTCCTGGAGATTAAA |
| *Danio rerio* | α-subunit | ATGGCGGCTTATCGTAGCGCGGCAGGCTTCCAACGTGTACTCCACGCGGTCAAGCATGTT TGCTTCAAGCAGCGTTGTGGCGTTATCATTTGCCGTTATTCCACCGTAATCGAGCCGAAC GACAAAACCTTCGACAAGATCCTCATCGCGAATCGTGGTGAAATCGCTTGCCGTGTTATC AAGACGTGCCGTAAAATGGGTATCAAAACCGTTGCCGTACATTCTGACGTAGACAGCTCT GCAGTTCACGTTAAGATGGCAGACGAAGCGGTTTGCGTTGGCCCTGCGCCGACCTCTAAA AGCTACCTGAACATGGACGCAATCATGAACGCGATCAAACTGACCGGTGCGCAGGCGGTT CACCCAGGCTACGGCTTCCTGTCTGAAAACAAAGAATTTGCCAAACGTCTCGCGGCTGAG GGTGTGACGTTCATCGGTCCGGACACCCACGCCATTCAGGCGATGGGTGATAAAATCGAA TCTAAGCTGATCGCCAAGGCGGCCAAGGTAAACACCATTCCAGGTTTCGATGGCGTCGTT AAGGACGCCGAAGAGGCAGTAAAAATCGCGGGTGAAATCGGTTATCCGGTCATGATCAAA GCGAGCGCAGGTGGCGGTGGTAAGGGTATGCGTATTGCGTGGAATGATGAAGAAACCCGT GAGGGTTTTCGCTTCTCCTCTCAGGAGGCTGCGTCCTCTTTCGGCGACGACCGTCTCCTG ATCGAAAAATTCATTGACAACCCGCGTCACATCGAGATTCAAGTACTGGCGGATAAGCAT GGCAATGCACTCTGGCTGAATGAGCGTGAGTGCTCTATCCAGCGCCGTAACCAAAAGGTT GTTGAGGAGGCGCCGTCTACCTTTCTCGACCCGGATACTCGCCGCAAGATGGGCGAGCAG GCCGTTTCTCTCGCAAAGGCTGTCAAATACTCTTCTGCGGGTACGGTTGAGTTCCTCGTG GACAGCAAGAAGAACTTCTACTTCCTGGAGATGAACACCCGCCTCCAGGTGGAACATCCG ATCACCGAATGCATCACTGGTCTGGATCTGGTTGAGCAGATGATCCGCATCGCTAAAGGC AACAAGCTCCAGCACAAACAATCTGACATCCCGATTAACGGTTGGGCTATTGAGTCCCGC GTTTACGCGGAGGACCCGTACAAATCCTTCGGTCTGCCGTCCATTGGCCGTCTGTCCCAG TACCAGGAGCCGCTGGACCTGGATAATGTTCGTGTAGATAGCGGCATCCAAGAGGGCAGC GACATCAGCATTTACTACGACCCGATGATCAGCAAGCTCGTCACGTATGGTAAGACGCGT GAGGAAGCTCTCAAAAAGATGGAAGAAGCCCTCGACAACTACGTTGTACGTGGTGTCACT CACAACATCCCACTGCTGCGCGAGATTATTGTGCATCCACGTTTTGTGAGCGGCGATATT TCTACGAAGTTCCTGCCGGAGGTGTACCCAGACGGTTTCAAAGGTCATATGCTCACGGCT GGCGAACGCCAAGAGCTGCTGGCCACGGCGGCAGCGCTCTACACCGCGGCACAACTGCGT TCTCAGCGTTTCCTCGGTGACCTCCGTGTTTCTTGCGTTCCGGCGGAATCTAGCGATTGG GAACTGTGCGTTGAACTGGAAAAGAGCGTGCACATGCTCGGCGTATCTCGCTCTGGTAAC AAGTACACCGTGGAGATTGACGGCGAAAAGGTGCAAGTCTCCGGCGAATGGAACCTGGCG TCTGCGCTGCTGTCTGTTACTATCAACGGTAAGCACCGCACGCTGCAATGCCTGTCTCGT ACGGCTGCCGGTGAGATCATCCTGCAGTACCTCGGTACGTCTTTCAAGCTGCGTGTCCTC TCTAAACTGGCCGCGTCTCTGTCTAAACACATGCCAGAGAAGGTACCGGAAGACACCTCT TCTATCCTCCGTTCCCCGATGCCGGGTAGCGTAGTCGCCGTTAGCGTTAAACCAGGTGAC ACGGTTGCGGAAGGTCAGGAAATCTGCGTGATCGAGGCAATGAAGATGCAGAACTCTATG ACCGCAGCCAAAACTGCGAAAGTTAAGTCTGTTCACTGCAAAGCGGGCGACACCGTTGGT GAAGGCGACCTCCTCGTTGAGCTGGAA |
| *Homo sapiens* | α-subunit | ATGGCTGGTTTTTGGGTAGGCACTGCTCCGCTGGTTGCTGCTGGTCGTCGTGGCCGTTGG CCTCCACAGCAACTGATGCTCTCTGCCGCTCTGCGTACCCTGAAACACGTTCTGTACTAC TCTCGCCAATGCCTGATGGTAAGCCGTAATCTCGGTTCCGTCGGTTATGACCCGAACGAA AAGACGTTCGACAAAATCCTCGTCGCGAACCGTGGTGAGATCGCTTGCCGCGTAATCCGT ACGTGCAAAAAGATGGGTATCAAAACGGTCGCGATTCACTCTGACGTGGATGCGTCTTCC GTTCACGTTAAAATGGCGGACGAAGCTGTTTGCGTTGGTCCGGCACCAACCTCTAAGTCT TACCTGAACATGGACGCGATCATGGAAGCAATCAAAAAAACTCGTGCACAAGCGGTTCAC CCGGGCTATGGTTTCCTCTCTGAGAACAAAGAATTCGCGCGCTGCCTCGCCGCTGAAGAC GTAGTATTCATCGGTCCTGACACGCATGCTATTCAGGCCATGGGTGATAAAATCGAAAGC AAGCTCCTCGCGAAAAAAGCCGAAGTAAACACGATCCCTGGCTTCGACGGCGTCGTGAAA GATGCCGAAGAAGCAGTACGCATCGCGCGTGAGATTGGCTACCCAGTTATGATCAAAGCG AGCGCTGGTGGCGGTGGTAAAGGTATGCGTATCGCGTGGGACGACGAAGAAACCCGTGAT GGTTTTCGTCTCAGCAGCCAAGAGGCCGCGTCCTCTTTTGGTGACGACCGTCTGCTCATT GAAAAGTTTATCGACAACCCGCGTCACATTGAGATTCAAGTTCTGGGCGACAAACATGGT AACGCGCTGTGGCTCAATGAACGCGAATGTTCTATCCAGCGTCGCAACCAAAAAGTTGTT GAGGAGGCCCCGAGCATTTTCCTCGATGCGGAGACTCGCCGTGCGATGGGCGAACAGGCA GTGGCCCTGGCCCGTGCGGTTAAATACTCTTCTGCGGGCACCGTGGAGTTTCTCGTCGAC AGCAAGAAGAACTTCTATTTCCTGGAGATGAACACGCGTCTGCAGGTTGAGCACCCTGTA ACGGAATGCATCACCGGTCTGGACCTGGTTCAAGAAATGATTCGTGTTGCGAAAGGTTAC CCTCTCCGTCACAAGCAGGCGGATATTCGTATTAACGGCTGGGCCGTAGAATGTCGCGTG TACGCGGAGGACCCGTACAAATCTTTCGGCCTGCCGTCTATCGGTCGCCTCTCTCAATAT CAGGAGCCGCTCCACCTGCCGGGTGTGCGCGTAGACAGCGGCATCCAGCCAGGTTCCGAC ATCAGCATCTACTACGACCCGATGATCTCCAAGCTGATCACGTATGGCTCTGATCGCACC GAAGCGCTGAAACGCATGGCCGATGCGCTCGATAACTATGTCATCCGTGGCGTAACCCAT AACATTGCGCTCCTCCGTGAAGTTATCATCAACTCCCGCTTCGTAAAGGGCGATATCAGC ACGAAATTCCTCAGCGATGTTTATCCGGACGGCTTCAAGGGTCACATGCTGACCAAGTCC GAGAAAAATCAACTGCTGGCAATCGCCAGCTCTCTCTTTGTGGCGTTCCAGCTCCGTGCG CAGCATTTCCAGGAGAACTCTCGTATGCCGGTTATCAAGCCGGACATCGCCAACTGGGAG CTGTCTGTAAAGCTGCACGACAAAGTTCACACTGTGGTGGCCAGCAACAACGGCAGCGTA TTTAGCGTGGAAGTCGACGGTTCTAAGCTCAACGTTACGTCTACGTGGAACCTCGCCTCC CCGCTGCTCAGCGTTAGCGTTGACGGCACCCAGCGTACGGTGCAATGTCTCTCCCGTGAG GCCGGTGGCAATATGTCCATCCAATTCCTGGGCACGGTGTACAAAGTCAACATCCTGACT CGTCTGGCCGCCGAACTGAACAAGTTCATGCTCGAGAAAGTGACCGAGGACACGAGCTCT GTCCTGCGTTCTCCGATGCCAGGCGTTGTAGTTGCGGTTTCTGTGAAGCCTGGCGACGCG GTTGCGGAAGGTCAGGAGATCTGTGTGATCGAGGCGATGAAGATGCAGAACAGCATGACG GCGGGTAAAACTGGTACGGTCAAGAGCGTGCACTGTCAGGCAGGCGACACCGTAGGTGAA GGTGACCTCCTGGTTGAACTCGAA |
| *Methylobacterium extorquens* | α-subunit | ATGTTTGACAAGATCCTGATTGCCAATCGCGGTGAAATTGCGTGCCGTATCATCAAAACG GCGCAAAAGATGGGTATCAAGACCGTTGCGGTTTATTCTGACGCCGACCGTGATGCAGTC CATGTGGCAATGGCAGATGAGGCTGTACACATCGGTCCGGCACCAGCGGCGCAGTCCTAC CTGCTGATCGAGAAAATCATCGACGCCTGTAAGCAAACGGGTGCGCAGGCGGTCCACCCG GGCTACGGTTTTCTCTCCGAGCGCGAATCTTTCCCAAAAGCCCTGGCTGAGGCCGGTATC GTTTTCATTGGTCCGAATCCGGGTGCCATCGCAGCTATGGGTGACAAAATCGAGTCTAAA AAAGCTGCGGCAGCAGCAGAAGTTAGCACTGTCCCAGGTTTCCTGGGTGTAATCGAATCT CCGGAACATGCGGTAACCATTGCTGACGAAATCGGTTACCCGGTTATGATCAAGGCGTCT GCTGGTGGCGGTGGTAAGGGTATGCGTATTGCCGAATCTGCGGACGAGGTAGCCGAAGGC TTCGCCCGTGCGAAAAGCGAAGCGTCTTCTTCCTTCGGTGACGATCGCGTGTTCGTGGAG AAATTCATTACCGACCCGCGTCACATCGAAATCCAGGTTATTGGCGACAAGCACGGCAAT GTTATTTACCTCGGTGAACGTGAATGCTCTATCCAGCGCCGCAACCAAAAGGTTATCGAA GAAGCGCCGAGCCCACTGCTGGATGAAGAAACTCGTCGCAAAATGGGCGAACAAGCTGTT GCGCTGGCCAAGGCCGTTAATTACGATTCTGCGGGCACCGTTGAATTCGTTGCCGGTCAG GACAAAAGCTTCTACTTCCTCGAGATGAACACTCGTCTGCAAGTGGAGCATCCAGTGACT GAAATGATTACGGGCCTGGATCTGGTTGAGCTGATGATTCGTGTTGCAGCTGGTGAAAAA CTCCCGCTGTCTCAGGACCAGGTAAAGCTGGACGGTTGGGCTGTAGAATCCCGCGTGTAC GCGGAAGACCCTACCCGTAACTTCCTGCCGTCTATCGGCCGTCTGACCACCTACCAGCCG CCAGAAGAGGGTCCACTGGGTGGCGCTATCGTTCGTAACGACACTGGTGTGGAGGAAGGC GGCGAGATTGCTATTCACTATGACCCGATGATCGCGAAGCTGGTTACTTGGGCGCCTACT CGCCTCGAAGCGATCGAGGCTCAAGCGACCGCACTCGACGCATTCGCAATTGAGGGTATC CGCCACAACATCCCGTTCCTGGCCACGCTGATGGCCCACCCTCGTTGGCGCGACGGTCGT CTGAGCACTGGCTTTATCAAAGAGGAGTTCCCGGAAGGTTTTATTGCCCCAGAGCCTGAA GGTCCGGTTGCCCATCGCCTGGCAGCCGTAGCAGCGGCCATCGACCATAAACTGAACATC CGTAAACGTGGTATTAGCGGTCAGATGCGTGACCCAAGCCTGCTCACTTTCCAACGTGAG CGTGTTGTTGTTCTGTCTGGTCAGCGTTTCAACGTTACCGTGGATCCGGACGGTGATGAC CTGCTCGTTACCTTTGACGACGGTACGACCGCGCCTGTTCGTAGCGCTTGGCGTCCTGGC GCACCGGTATGGAGCGGTACTGTAGGTGATCAAAGCGTAGCGATTCAGGTACGTCCACTC CTCAATGGCGTGTTTCTGCAGCACGCAGGTGCGGCTGCTGAAGCGCGTGTGTTTACCCGC CGTGAAGCGGAACTCGCGGACCTCATGCCAGTGAAAGAGAACGCCGGCTCCGGCAAACAG CTCCTCTGCCCGATGCCGGGTCTGGTAAAACAGATCATGGTTTCTGAGGGCCAAGAGGTC AAGAATGGTGAACCTCTGGCCATTGTTGAAGCTATGAAGATGGAAAACGTCCTCCGCGCA GAGCGTGATGGTACTATTTCTAAGATTGCAGCCAAGGAGGGCGACTCTCTCGCAGTAGAC GCGGTTATCCTGGAATTTGCG |
| *Myxococcus fulvus* | α-subunit | ATGCCGAAGATCCGCAAGGTTCTGGTCGCGAACCGTGGCGAGATTGCTATCCGTGTGATG CGTACGTGTAAAGAGCTCGGCATTGCGACCGTTGCCGTTTACTCCGAAGCGGATCGTTCC GCTCTCCATGTTCGTACCGCCGACCAGGCAATTTTCGTTGGCCCTCCGCCGTCTCGTGAA TCTTATCTGGTTCAGCAGCGTATCCTCGACGCCGCTAAACAGGCTGGCGCAGATGCGATT CATCCGGGCTATGGTTTCCTGTCTGAAAACGCGTCTTTCGTTCGTGCGTGCGAGTCTGCC GGCCTGACCTTCATTGGTCCTCCAGCTTCTGCTATGGACAGCATGGGCGAAAAGACCCGT GCTCGTGCCAATATGATCAAAGCGGGTGTTCCTGTTGTCCCGGGCACGACCGAACCTATT GCTACTGTTGAGGAAGCACGCGCTTACGTGGAGAAGATCGGCTTCCCGGTAATGCTGAAG GCGGCAGGTGGTGGTGGTGGTAAAGGTATGCGCAAAGTGGAACGCATGGAGGATTTCGAG TCTAGCTGGCGTTCTGCCAAGTCTGAGGCCCTCAATGCGTTTGGTAACGATGCGGTCTAC ATCGAGAAATACCTCGAAAAACCGCACCACGTTGAGATCCAAGTGTTTGCGGACCAGTAC GGTAACACCATCCACCTCAACGAACGTGAGTGCTCCGCCCAGCGTCGTCACCAGAAAGTT GTGGAAGAAACCCCGTCTCCGATCCTCACGCCGGAACTGCGTGCAAAAATGGGTGAAGTC GCCGTTAAGGCCGCGAAAGCGGTTAACTACGTTGGCGCTGGCACCGTTGAATTCCTGGTG GACGTTCACCGCAACTTTTACTTCCTCGAGATGAACACTCGCCTCCAGGTTGAGCATCCG GTGACTGAATGGGTTACCGGTCTGGACCTGGTTGCGATGCAGATCCGTGCGGCCGAGGGT GAAAAACTCGGCCTCACGGAAGCCCCACAGCCTAACGGTCACTCTATCGAAGTTCGTGTT TACGCGGAGGATCCGAGCCGTAACTTCATGCCGTCCCCGGGTAAAATCACCTACCTGCGT GTTCCGGGTGGTCCAAATGTCCGTGATGATTCTGGCGTCTTCCCTGGTTACACCGTTCCG AACGTTTACGACCCGATGATCTCTAAGCTGTCTGTTTGGGCCCCTACGCGCCGTGAAGCC ATCGCTCGCGCCCAACGCGCGCTGTCCGAATACGTGGTTAAGGGCATCACTACCAACATC CGCTACCTCAAGGCGATCCTGGCGAATCCGGAGTTCATCGAAGGTGACTACGACACCTCT TTCCTCACCCGTCAGCATGACGCTCTGCTGGGTAAAGAAGACCCAAAACTCTCTGAAATG GCACTGCTCGCAGGTGTTGTTCACGCGTATCAGCGCGATCAGAAACGTGCTAAGACGCTG CCGGGTGCCGGTGGCGGTCAGGGCGGTGGCAATGGCGGTGTATCTCCGTGGAAACTCGCG CTCCGCACTCGTCGC |
| *Paracoccus sp.* | α-subunit | ATGTTTAAGAAGATCCTCATCGCGAACCGTGGTGAAATCGCGTGCCGTGTTATCAAAACC GCGAAGAAAATGGGCATCCAGACCGTTGCGGTTTATTCTGACGCTGATCGCAACGCCCTG CATGTAAAAATGGCTGATGAGGCCGTACACATTGGTCCGTCCCCTGCGAACCAGTCCTAC ATTGTAATCGACCGTATCATGGAAGCGATCCGTCAAACTGGTGCCGAAGCTGTTCATCCG GGCTATGGTTTCCTCTCTGAAAACATGAAATTCGCGGAGGCTCTGGAAAAAGAGGGCGTA GCGTTCATCGGCCCTCCTTCTCCGGCGATCGAAGCTATGGGTGATAAGATTACCTCTAAG AAACTGGCACAAGAAGCCGGTGTTTCTACCGTTCCAGGTTATATGGGCCTGATTGCCGAT GCTGACGAGGCAGTCAAAATTTCTAAGGAGATCGGCTTCCCAGTGATGATCAAGGCGTCC GCTGGCGGTGGTGGCAAAGGTATGCGTATTGCGTGGAACGAACAGGAGGCACGTGAAGGC TTTGAAAGCTCCCGTAACGAGGCTGCCGCGTCTTTCGGCGACGACCGCATCTTTATTGAG AAGTTCGTGACTCAACCGCGCCATATCGAAATCCAAGTTCTGGCCGATCAACATGGTAAC TGTGTATACGTTCACGAGCGTGAGTGTTCTATCCAGCGTCGTAATCAAAAAGTGATTGAG GAAGCGCCTAGCCCGTTCCTGGACGAAGCCACTCGTAAGGCCATGGGCGAACAAGCAGTT GCGCTGGCCAAAGCGGTGGGTTATGCGAGCGCAGGTACTGTCGAATTCATCGTGGACGGT CAGAAAAACTTCTACTTTCTCGAAATGAACACGCGTCTGCAGGTGGAACACCCAGTAACC GAGCTGATCACCGGTATTGATCTGGTGGAGCAGATGATCCGTGTGGCAGCTGGTGAGCCG CTCCCATTCCGCCAAGAGGACCTGAAAATCAACGGTTGGGCAATCGAATCTCGTCTGTAC GCCGAGGACCCGTACCGTAACTTCCTGCCGTCTATCGGCCGTCTGACCCGTTATCGCCCA CCACGCGAAACCGCTGCCGGTCCTATGCACGAAGCGGGTAAATGGGTTCCGGCACACCCT GGTGACCAGCCGCCTGCAGCAGCCGCTGGTGTTCGTAATGACACTGGCGTCTACGAAGGT GGCGAAATCTCTATGTACTACGACCCGATGATCGCCAAGCTGTGCACCTGGGCTCCGACC CGTGCTGAAGCCATTGAACAGATGCGCCTCGCACTCGATGAGTTCGAAGTTGAAGGTATC GGTCATAATCTGCCGTTTCTCTCTGCGGTCATGGACCATCCGAAGTTTGTATCCGGTGAC ATCTCCACGGCATTCATTGCGCAGGAATACCCAGAGGGTTTTCTCGGTGCGACCCTGCCT GACACCGAAATCATTCGTGTTGCGGCAAGCGCTGCGGCGATGCACCGTGTAGCGGAAATC CGTCGTGCGCGTATCTCTGGTCGTCTGGACAACCATGAACGTCACGTTGGCGAGCATTGG GTTGTTTTCGTAGCCGGTAAAGAAATCCCGGTACGTATCACCGCGGATCGTCAGGGTTCC ACTGTGGAAATCGACGGCTCTAAACTGCGTGTTGAATCTGATTGGCTGCCTGGTCAGTCT CTGGCACGCCTGTCTGTAGATGGCACCCCGCTCGTTATGAAAGTCGATCGTCTGCCATCC GGTTTCCGTCTCCGCGTACGTGGTGCGGATCTCAAGACCTACGTTCGTCGTCCTCGCGCA GCTGAGCTCGCTCGCCTGATGCCAGAAAAGCTGCCTCCGGACACTTCTAAATTCCTCCTC TGCCCGATGCCTGGCCTCGTAGTTCGTATTAACGTTGAAGCAGGTGATGAAGTTCAAGAA GGTCAAGCCCTGGCGACCGTCGAGGCGATGAAGATGGAGAATATCCTGCGTGCGGAAAAG AAGGCTGTTGTAAAATCTGTGAATGCAAAGCCGGGTGAATCCCTCAAAGTTGACGACGTT ATCATTGAATTTGAA |
| *Propionibacterium acnes* | α-subunit | ATGGATCGTACCATCAAGCGTGTGCTGGTTGCGAACCGTGGCGAAATCGCGGTGCGTATC ATCCGTGCTGCGAAAGACGTAGGCATCACCTCTGTCGCGGTTTACGCCGATTCTGATTCC GAGGGCCTCTTTGTTAAACTGGCGGATGAGGCGTTTGCGCTGAACGGTGTTACCCCGGCT CAGACTTATCTGGATGTTGATAAAATCCTGGACGTAGCGCGTCGTGCAGAAGTTGATGCA ATCCACCCGGGCTATGGTTTCCTCTCTGAAAACGCGGATTTTGCCCAAGCGGTCATTGAT GCGGGCTTCCTGTGGATCGGCCCACCTCCACACGCCATTCGCGTTCTCGGTGATAAAGTT CAGGCCCGTCACATCGCGCAGAAAGTGGGTGCCCCGCTGGTCCCAGGTACCGCGGATCCT GTAGACGATCCGGAGGAGGTTGTGCAGTTCGCTAAGGAACATGGTCTGCCGCTGGCTATT AAGGCCGCATTCGGTGGCGGTGGTCGCGGCCTCAAGGTTGCGCACTCTATGGACGACATC ACGCCGCTCTACGAAAGCGCTGTTCGTGAGGCGGTGACCGCCTTTGGCCGTGGTGAGTGT TTCGTCGAGCGTTACCTCGATAAACCACGTCACGTGGAGACGCAGTGCATTGCGGACCAT TACGGCACGGTACAGATTATCTCTACCCGTGACTGCTCTCTGCAGCGTCGTCATCAAAAG CTGGTCGAGGAAGCGCCTGCGCCTTTTCTGTCTGACGAGCAACTGACTCGCCTGTACGAA TCCAGCCGTGCAATTCTGAAGGAGGCCGAATATGTTGGTGCGGCGACGTGTGAATTTCTC GTTGGTCAGGACGGCACCATTTCTTTCCTGGAGGTCAACACCCGCCTCCAAGTTGAGCAC CCGGTTTCCGAAGAAGTAGCAGGCGTTGATCTCGTTCAGATGATGTTCCACGTTGCACAG GGTGGCCATCTCACCCCGGAAGACCCGCAACTGCGTGGTCACTCTTTCGAATTTCGTATT AACGCAGAGGATCCAGGTATGGGTTTCATGCCAGCACCTGGCACGCTCACTTCTTGGCAG CCACCATACGGTCCGGGCGTCCGCATGGATGAAGGCTACCACTCTGGTATGACCGTTCCT GGTGCCTTCGATTCTCTCATCGGTAAACTCATCATCACCGGCGACTCTCGCGAACAGGCT CTCGCACGCGCAGCCCGTGCGCTCGATGAAATCGTTATCGACGGTATGCCGACCGTCATC CCGTTCCACCAGGCAGTTGTTCACGACCCAGCATTCACGGCAGCTGATGGCTGTTTCGGT GTGTTCACCGACTGGATCGAAACCGAATTTGACAACAAGATTGAACCGTACACCGGTTCT CTGGGCGAATCTGCCAACTCCGAACCTCCGCGCGAGGTGGTAGTTGAAGTCAATGGCAAA CGTGTCGAAGTTCGTATCCCGGCGTCTCTCCATGGTGCGCGCCAAGTTCCATCTACCAAA TCCCACCGTCCGACTCGTCGTAATCGCGCTGGCCGCCGTGCGGCAGCGACCGGCAACTCT GTAGCTTGCCCGATGCAGGGTACTATTATCAAAGTTAACGTGGTGGAGGGTGAGGAAGTG AATGAAGGTGATCAGATCGTCGTTATGGAAGCAATGAAGATGGAGCAACCGATTGCCGCG CATCGCTCTGGCATCGTCCGTAACCTGAATGCGATGGTTGGTTCTACCGTTTCTTCTGGC GAAGTAGTTTGCGAGATTGTGGACGAG |
| *Rhodobacter sphaeroides* | α-subunit | ATGCATCTGCGTGACCGTCCTGGCTTCGGCCCAGGTGGCCCTTTCCGTTTTCGTGCACTC CCACGCGCTGCAGACAAGGGCACCATGATGTTCAAGAAGATCCTCATCGCGAATCGTGGC GAAATCGCCTGTCGCGTGATCAAAACGGCTCGTAAAATGGGCATCCAAACCGTGGCCGTT TACTCCGATGCGGATCGTAATGCACTGCACGTGTCTATGGCCGACGAAGCAATCCACATC GGCCCTCCTCCGGCAAACCAGAGCTACATCGTCATCGACAAGATCATGGAGGCCATCAAA GCGAGCGGCGCTGAGGCAGTACACCCGGGCTACGGTTTCCTGTCTGAACGCATGGACTTC GCTGCCGCTCTCGAAGCAGCTGGCGTTGTATTCATTGGCCCACCGAGCGGTGCAATTGAG GCGATGGGTGATAAGATCACCTCCAAAAAGCTCGCGAAGGAGGCCGGTGTGTCTACCGTC CCAGGCTACATGGGTCTGATCGCTGACGCCGATGAAGCAGTAAAGATCTCTAACGAGATC GGCTACCCGGTCATGATCAAGGCCTCTGCTGGTGGCGGTGGTAAAGGCATGCGTATTGCG TGGTCTGAAGCCGAGGTCAAGGAGGGTTTTGAGTCTTCTAAGAACGAGGCCGCGAACTCT TTCGGCGACGATCGCATCTTTATCGAGAAGTTTGTAACCCAGCCGCGTCACATCGAGATT CAGGTTCTGGCTGACAAACACGGTAACTGCGTGTACCTGCACGAGCGCGAATGTTCTATC CAGCGTCGTAACCAGAAAGTTATTGAAGAGGCGCCATCCCCGTTCCTGGATGAGGCCACT CGTAAGGCCATGGGCGAGCAGGCTTGCGCCCTGGCAAAAGCGGTTGGCTATGCCTCCGCA GGCACTGTTGAATTTATCGTGGACGGCCAGAAGAACTTCTACTTCCTCGAGATGAACACC CGTCTCCAGGTCGAACATCCGGTTACCGAACTGATCACCGGTATCGACCTCGTAGAGCAA ATGATTCGTGTAGCTGCCGGTGAAAAACTGCCGTTCCAGCAATCTGACCTGAAGATCAAC GGTTGGGCAATGGAGTCTCGTCTGTATGCCGAGGACCCATATCGTAACTTCCTGCCGTCT ATCGGCCGTCTCACTCGTTATCGTCCTCCAGTTGAATCTGTTACCCCTACGTCTGTGGTT CGTAACGACACGGGCGTTTACGAAGGCGGCGAGATTAGCATGTATTATGACCCGATGATC GCCAAACTGTGTACGTGGGCTCCGACGCGCGAAGCCGCGATCGAAGAAATGCGTCTCGCC CTCGACACCTTTGAGGTTGAAGGTATTGGTCACAATCTCCCTTTCGTTGGCGCCGTTATG GACCATCCGCGCTTCGTTAAGGGCGACATCACTACGGCATTTATTGCGGAAGAATACCCG GACGGTTTTCAAGGTGCGGTACTGGACGAACCGACCCTGCGTCGTGTTGCCGCAGCCGCC GCTGCCATGAATCGCGTTGCGGAGATCCGTCGTACCCGTATTTCTGGCACGATGAATAAC CACGAACGTCACGTTGGTGTTGATTGGGTAGTAGCGCTCCAGGGTGAGAGCTATCACGTT TCTATCGCTGCGGACCGTGAGGGTAGCACCGTATCTTTCTCTGACGGCTCTTCTCTGCGC GTTACCAGCGACTGGACCCCTGGCCAACCTCTCGCTAGCCTCATGGTCGACGGTCGTCCG CTGGTGATGAAAGTTGGTAAAATTCCGATGGGCTTTCGTCTGCGCCTGCGCGGTGCTGAC CTCAAGGTTAATGTCCGTACTCCACGTCAAGCAGAACTGGCCCTGCTGATGCCTGAGAAG CTCCCACCGGATACCTCTAAATATCTCCTGTGCCCAATGCCTGGCCTGGTCGTGAAGATT AACGTGGCGGAGGGTGACGAGGTACAGGAAGGTCAGGCCCTCGCGACGGTGGAAGCGATG AAAATGGAAAATATCCTCCGTGCCGAGCGCCGTGGTACCGTAAAGAAAATTGCTGCTGCA CCTGGTGCCTCTCTCCGCGTAGACGACGTGATTATGGAATTCGAA |
| *Sinorhizobium fredii* | α-subunit | ATGACCCGCCAACGTGAGCTGCGCGAAAAACGCATGTTCAAGAAAATTCTCATTGCGAAC CGTGGTGAAATCGCGTGCCGTGTTATCCGCACGGCCAAAAAACTGGGCATCGCCACTGTA GCGGTGTATTCTGACGCAGACCGCGACGCAATGCACGTACGCCTCGCTGACGAGGCCGTA CATATTGGTCCGTCCCCGAGCAGCCAGTCTTATATCGTAATCGATAAGATCCTGGAAGCT ATTCGCAAGACGGGCGCAGATGCGGTGCATCCGGGTTATGGTTTCCTGTCCGAAAACGCG GCCTTCGCAGAAGCCCTGGAGAAAGAAGGTGTGGCATTCATCGGTCCGCCAGTAAAGGCT ATTGAAGCCATGGGCGACAAAATTACTTCTAAAAAGCTCGCCGCTAAAGCCGGTGTGAGC ACGGTCCCTGGTCACATGGGTCTGATCGAAGACGCCGACGAAGCGGCTCGCATTGCTTCT AGCATTGGCTTTCCGGTTATGATCAAGGCCTCCGCCGGTGGCGGTGGTAAGGGTATGCGT ATCGCCTGGAACGAGCGCGAGGCCCGTGAAGGCTTCCAATCCAGCAAAAACGAGGCTAAA TCTTCCTTTGGCGACGATCGCATCTTTATTGAGAAGTTCGTGACTGAGCCTCGCCACATC GAAATCCAAGTTCTGGGTGACAAGCATGGTAATACTCTGTATCTCGGCGAACGCGAATGT TCTATTCAACGCCGCAATCAAAAGGTGATCGAGGAGGCCCCTTCTCCATTTCTCGACGAA ACCACTCGTCGTGCGATGGGTGAACAAGCGGTCGCACTCGCGAAAGCGGTTGGCTACCAC TCCGCTGGCACTGTTGAATTTATCGTGGACGCGGAACGCAACTTTTACTTCCTCGAGATG AACACCCGTCTGCAGGTGGAACATCCTGTCACGGAGCTGGTTACTGGTCTGGACCTGGTA GAGCAAATGATCCGTGTCGCGGCTGGTGAGAAGCTGTCTTTCACGCAAGATGACGTAAAG CTGGAAGGCTGGGCCATCGAATCTCGCCTGTACGCTGAAGATCCGTACCGCAATTTCCTC CCAAGCATCGGCCGTCTCACCCGCTATCGCCCTCCGGAAGAGGGTCGCCAGGACGACGAT ACTGTAATCCGTAATGATACCGGCGTGTTCGAAGGTGGCGAAATCTCCATGTACTACGAC CCTATGATTGCCAAGCTGTGCGCATGGGGTCCGGATCGCGCCCAGGCAGTAGAGGCTATG GCCAAAGCGCTCGACGCTTTTGAAGTGGAGGGTATCGGCCACAATCTGCCGTTTCTGAGC GCCGTGATGCAGCAAGAGCGTTTCCGCGAAGGTCGCCTCACGACTGCCTATATCGCCGAA GAGTTCACGGACGGCTTTCAGGGCGTGACCCCTGATGAGGCTGCGGCTATGAAACTGGCA GCTGTAGCCGTTACCGTAAATCAGGCGCTCCAGGAACGTGCTAGCCAGATCAGCGGCACC ATTGGTAACCACCGTCGTATCGTAGGTCATGATTGGGTAACCTCCCTGGCTGAACGTGAA TTTCAAATGACTTCCGGTGTCTCTGCTGACGGCGCATACGTTCGCTTTGCGGACGAGCGT GCAATTAGCGTAGCATCCGACTGGGTACCTGGTCGTACCCTGGCCACTTTTAACATCGAC AACCAGTCCATGGCAGTGAAAGTGGATCTCGTAGGCACGGGCATTCGTCTGCGCTGGCGT GGTATTGATGTGGTTGCTCGTGTACGTAGCCCACGCGTTGCGGAGCTCGCACGTCTCATG CCAAAGAAACTCCCACCGGACACCTCTAAGATGCTGCTGTGCCCTATGCCAGGTGTCGTA ACGTCCATCACCGTCAAGGCAGGTGACACCGTTGAAGCTGGCCAATCTCTGGCCGTAGTC GAGGCCATGAAAATGGAAAACATTCTCCGTGCTGAGAAACGCGCGACTGTTAAACGTGTT GCCATCGCCGCAGGTGCCTCTCTCGCTGTAGATGAGCTGATCATGGAATTCGAA |
| *Streptomyces coelicolor* | α-subunit | ATGCGTAAAGTTCTGATTGCAAACCGTGGCGAAATCGCGGTGCGCGTAGCACGCGCCTGC CGTGATGCAGGCATCGCGTCCGTTGCCGTGTACGCCGACCCGGATCGCGACGCGCTGCAT GTGCGTGCCGCCGATGAAGCTTTCGCTCTGGGCGGTGACACGCCTGCGACTTCCTACCTC GACATCGCCAAGGTTCTGAAAGCAGCCCGTGAATCTGGTGCGGATGCGATCCACCCTGGT TACGGCTTTCTCTCCGAAAATGCGGAGTTTGCTCAGGCGGTCCTGGACGCTGGCCTGATC TGGATCGGCCCACCTCCTCACGCCATCCGTGATCGCGGCGAAAAGGTGGCTGCACGCCAC ATCGCGCAACGTGCCGGTGCCCCGCTGGTGGCCGGCACCCCAGACCCGGTTAGCGGTGCT GATGAGGTGGTAGCGTTTGCCAAAGAGCATGGTCTGCCAATTGCCATCAAGGCGGCCTTT GGCGGTGGTGGTCGTGGCCTCAAGGTCGCCCGTACGCTCGAAGAAGTCCCTGAGCTCTAT GATTCCGCTGTACGCGAAGCTGTCGCAGCCTTCGGCCGTGGTGAATGCTTTGTTGAACGT TATCTGGACAAGCCTCGTCACGTCGAAACGCAGTGTCTCGCGGACACCCACGGTAATGTA GTCGTGGTTTCTACTCGCGACTGCTCTCTGCAGCGTCGTCATCAAAAGCTGGTTGAGGAA GCTCCGGCTCCGTTCCTCAGCGAAGCGCAGACGGAACAACTGTACTCTTCTAGCAAAGCA ATCCTCAAAGAGGCGGGCTATGGTGGCGCAGGCACGGTAGAATTTCTCGTGGGCATGGAC GGTACTATCTTCTTCCTGGAGGTTAACACGCGTCTGCAGGTTGAACACCCTGTCACTGAA GAGGTAGCCGGTATCGATCTCGTGCGTGAAATGTTTCGCATCGCCGACGGTGAAGAACTC GGCTACGATGATCCAGCGCTCCGTGGTCACTCCTTTGAATTCCGTATCAATGGCGAGGAC CCAGGTCGCGGTTTTCTCCCTGCCCCTGGCACTGTGACTCTGTTCGATGCGCCAACGGGC CCTGGCGTACGTCTCGACGCCGGTGTGGAGTCTGGTTCTGTCATTGGTCCTGCGTGGGAT TCCCTCCTGGCTAAACTGATTGTAACCGGTCGTACCCGTGCCGAGGCGCTCCAGCGTGCT GCTCGCGCCCTCGATGAGTTTACGGTGGAAGGCATGGCGACGGCTATCCCTTTCCATCGC ACCGTGGTACGTGACCCGGCGTTCGCCCCAGAACTCACCGGCTCTACCGACCCTTTCACT GTCCATACGCGTTGGATTGAGACGGAGTTCGTAAACGAAATCAAGCCATTCACGACCCCT GCCGACACCGAAACCGACGAGGAATCCGGTCGCGAAACGGTAGTAGTTGAAGTCGGTGGT AAACGCCTGGAAGTCAGCCTCCCATCTTCTCTCGGTATGTCTCTGGCTCGCACTGGTCTG GCAGCTGGTGCTCGTCCTAAACGTCGCGCAGCAAAAAAGAGCGGTCCAGCAGCTTCTGGC GACACCCTCGCCTCCCCTATGCAAGGCACTATCGTGAAAATCGCTGTCGAAGAGGGCCAA GAAGTGCAAGAAGGTGACCTGATCGTCGTACTGGAAGCGATGAAAATGGAGCAACCGCTG AACGCACACCGTTCTGGCACGATTAAAGGCCTGACGGCAGAAGTAGGTGCCTCTCTGACT AGCGGCGCAGCTATCTGCGAGATCAAGGAT |
| *Actinoplanes sp.* | β-subunit | ATGACCCTCCCGCCATCCTATGATCTCAACCGCACTGCTATGCTGGAACGTGTTGCCGAT CTCGAACGCCAACACGCAGTGGCGGTAGCCGGTGGTGGCCCGAAAGCCGTTGAGCGTCAT CACCGCCGTGGTAAACTGACCGCGCGTGAGCGCATCGAACTGCTGCTGGACGAAGACTCT GCGTTCCTGGAGCTGTCTACCCTGGCAGGTTGGGGCACTGACGTAACCGTTGGTGGCTCT GTAGTGACTGGCATCGGTGTCGTTGAAGGTGTAGAATGCATGATTGTTGCGAACGATCCG ACTGTTCGTGGCGGCTCTTCTAACCCGGTTACCGTAAAGAAATCTTTCCGTGCCGCGCAA ATCGCTGCCGAAAATCGTCTGCCAACGATCAACCTGGTTGAATCTGGTGGTGCCGACCTG CCTACTCAGAAAGACATCTTCATCCCGGGTGGTCGTCACTTCCGCGGTCTGACCGAAGCA AGCGCCGATCGCCGTCCGACCATCGCGCTGGTCTTCGGTAACGCGACCGCTGGCGGCGCA TACGTTCCGGGTATGTCTGATTACGTTGTTATGGTTGCAGGCGGTGCTAAAGTTTTCCTC GGTGGTCCTCCGCTGGTTAAAATGGCAACCGGTGAAGAATCCGATGACGAGAGCCTGGGT GGCGCCCAAATGCACGCACGTACCTCCGGCCTCGCGGACTTTCTGGCAGTAGACGAGACT GATGCTATCCGTATCGGCCGTCAAATCGTACGTCGTCTGAATTGGCGCAAGCTGGGTCCA CCTCCACGTGCGGGTTACGCGGAGCCAGTTCTCGACGCCGAAGAGCTGCTCGGCCTGATC CCTAGCGACCTGAAGGTTCCGTTCGACCCACGTGAAGTCGTATGGCGTATCGTTGACGGT TCCGAGTTCGACGAATTTAAGCCGCTGTACGGTACCTCTCTGGTCACGGGCTGGGCGCGT CTCCACGGTCACCCAATCGGCATCCTGGCGAATGCGCAGGGCGTTCTGTTTTCTGCGGAG GCGCAGAAGGCTGCGCAATTCATCCAACTCGCGAACCAAGCGGACACCCCTCTGCTGTTT CTGCACAATACGACGGGCTACATGGTTGGTAAGGAATACGAGCAAGGTGGCATCATCAAA CACGGTGCACAGATGATCAACGCGGTTTCTAACTCTCGTGTTCCTCACCTGTCCGTTGTC ATGGGTGCCTCTTACGGTGCTGGTAATTATGGCATGTCTGGTCGCGCTTACGATCCTCGC TTCATGTTCTCTTGGGTTGGTGCGAAGTCTGCCGTAATGGGTCCGGCGCAGCTGGCTGGT GTTGTATCTATCGTTAGCCGTCAGGCGGCAGCCGCAGCAGGCCGTCCTTTCGATGAGGAA GCAGACGCCCGCACTCGTGCTGCTATCGAAGCGCAGATTGAACGTGAATCTCTCGCGTAC TTCACTAGCGGTATGCTCTACGACGACGGCGTAATCGACCCTCGCGACACGCGTACCGTT CTGGGTATCTGCCTGTCTGTTATCCATAACGCGCCTGTTCAGGGTGCGCGTGGTTACGGC GTCTTTCGTCTG |
| *Bos taurus* | β-subunit | ATGGCAGCCGCGATGCGTGTTGCGGTAGCCGGTGCGCGCCTGAGCGTCGTTCTCCGCTCT CTCCGTGGTGCGGGTCGTAGCCTCTGCACCCAGCCAGTTTCTGTTAACGAGCGTATCGAG AACAAACGCCAGGCGGCACTGCTCGGCGGTGGTCAACGCCGTATTGACGCACAGCATAAA CGCGGTAAGCTCACTGCCCGTGAACGCATTTCTCTGCTGCTGGATCCGGGCTCTTTCGTG GAATCTGATATGTTCGTAGAGCATCGCTGCGCCGACTTCGGTATGGCTGCCGACAAAAAC AAATTCCCGGGTGACTCTGTCGTTACTGGTCGTGGTCGCATCAATGGTCGCCTCGTGTAC GTTTTCTCTCAGGACTTCACCGTTTTTGGTGGTTCTCTGTCCGGTGCCCATGCCCAAAAG ATTTGTAAGATCATGGACCAGGCGCTGACTGTTGGTGCGCCGGTTATTGGCCTGAACGAC TCCGGTGGTGCTCGTATCCAGGAAGGTGTAGAATCCCTGGCGGGTTACGCGGATATCTTT CTCCGTAATGTAACCGCTTCCGGCGTGATCCCGCAGATCTCCCTGATTATGGGTCCGTGT GCAGGTGGCGCTGTTTACTCCCCAGCGCTCACCGACTTTACCTTCATGGTTAAAGACACG AGCTACCTGTTCATCACTGGCCCAGACGTTGTTAAATCTGTCACCAACGAGGACGTGACC CAGGAGGAGCTGGGCGGTGCACGCACTCACACTACTATGTCTGGTGTGGCCCATCGCGCG TTCGAAAATGACGTAGATGCGCTCTGCAACCTCCGCGAATTCTTCAACTACCTGCCGCTG TCCAACCAGGACCCTGCCCCAGTTCTGGAGTGTCACGATCCATCTGATCGTCTCGTCCCA GAGCTGGACACCATCGTGCCACTGGAGTCTACCAAGGCGTACAACATGGTCGACATTATC CACGCGGTTGTAGACGAGCGCGAGTTCTTCGAAATCATGCCGAACTACGCGAAAAACATC ATTGTAGGCTTTGCGCGTATGAACGGTCGTACCGTTGGTATTGTTGGCAATCAGCCAAAA GTTGCGTCCGGTTGCCTGGACATCAATAGCAGCGTTAAGGGTGCCCGCTTCGTGCGTTTC TGCGACTCTTTCAACATTCCGCTGATCACGTTCGTCGATGTCCCTGGTTTCCTGCCGGGT ACTGCGCAAGAATACGGTGGCATTATTCGTCACGGTGCTAAACTCCTGTACGCGTTTGCC GAAGCAACGGTCCCGAAGGTGACCGTCATCACCCGTAAAGCGTATGGCGGCGCTTACGAC GTCATGAGCAGCAAACACCTCTGCGGTGACACCAATTATGCTTGGCCGACCGCGGAGATC GCTGTAATGGGCGCAAAAGGTGCTGTCGAGATCATCTTCAAAGGCCACGAGAACGTGGAA GCCGCACAGGCAGAGTACATCGAAAAATTCGCCAACCCGTTCCCTGCGGCAGTTCGTGGC TTCGTTGACGATATCATTCAGCCGTCTTCTACGCGTGCCCGTATCTGTTGTGACCTGGAC GTTCTGGCGTCTAAAAAAGTACAGCGTCCATGGCGTAAGCATGCCAATATTCCTCTG |
| *Chloroflexus aurantiacus* | β-subunit | ATGTCTATCATCCATTCCCATATCCAGCCGAACTCTCCGGACTTCCAGGCGAACTTTGCG TATCATCAATCCCTGGCTGCGGACCTCCGCGAGCGTCTGGCGCAGATTCGTCAAGGTGGT GGCGCCGAGCAACGTCGTCGTCATGAAGAACGTGGTAAGCTGTTTGTCCGTGACCGTATC GACACGCTGATCGACCCGGATAGCTCTTTTCTGGAAATCGGTGCACTCGCGGCCTACAAC GTCTACGACGAAGAGGTACCGGCAGCAGGTATTGTGTGCGGTATTGGTCGTGTTGCGGGT CGTCCGGTGATGATTATCGCGAACGACGCGACTGTTAAGGGCGGTACTTACTTTCCGCTG ACCGTCAAAAAGCATCTGCGTGCGCAGGAGATCGCCCGTGAGAATCGCCTCCCGTGTATC TATCTCGTTGACTCCGGTGGTGCATACCTCCCGCTGCAGTCTGAGGTGTTTCCGGACCGC GATCACTTTGGCCGTATTTTCTACAACCAGGCACAGATGAGCGCGGAAGGTATCCCGCAG ATCGCGTGCGTAATGGGTAGCTGCACGGCTGGCGGCGCTTATGTTCCTGCGATGTCCGAC GAGGTGGTTATCGTAAAGGGTAACGGCACGATCTTCCTCGGTGGTCCGCCGCTGGTTAAG GCGGCAACCGGCGAAGAAGTAACCGCAGAAGAGCTGGGCGGTGCGGACGTACATACGCGC ATTTCTGGCGTGGCAGACTACTTCGCAAACGACGATCGCGAGGCTCTGGCTATTGTTCGT GACATCGTTGCGCACCTGGGTCCACGTCAACGTGCAAACTGGGAGCTGCGTGACCCTGAG CCACCGCGCTACGATCCACGTGAAATCTACGGTATTCTGCCGCGTGATTTCCGCCAATCT TACGACGTGCGTGAGGTAATCGCACGTATTGTCGACGGTTCCCGTCTCCACGAATTTAAG ACCCGTTACGGTACCACTCTGGTTTGCGGTTTCGCGCACATCGAGGGTTTTCCAGTAGGC ATCCTCGCCAACAATGGCATTCTGTTCTCTGAATCCGCACTGAAAGGTGCCCACTTCATC GAACTGTGCTGTGCCCGCAATATTCCTCTCGTATTCCTGCAGAACATTACGGGCTTTATG GTTGGCAAGCAGTATGAAAATGGCGGCATTGCTAAGGACGGTGCGAAGCTGGTGACCGCC GTCTCTTGCGCCAACGTTCCGAAATTCACGGTGATCATTGGTGGTTCTTTCGGTGCTGGT AACTATGGCATGTGTGGTCGCGCCTACCAGCCGCGTCAACTCTGGATGTGGCCGAACGCG CGCATCTCTGTTATGGGCGGCACTCAAGCAGCGAATGTGCTCCTGACCATCCGCCGTGAC AACCTCCGTGCGCGTGGCCAAGACATGACGCCGGAAGAACAAGAGCGTTTCATGGCACCA ATCCTGGCCAAATATGAGCAGGAGGGCCACCCGTACTATGCGTCTGCCCGTCTGTGGGAT GACGGTGTAATTGATCCGGTCGAAACTCGTCGTGTACTGGCCCTGGGCCTCGCCGCAGCA GCTGAAGCTCCGGTACAGCCGACGCGTTTTGGTGTTTTCCGTATG |
| *Corynebacterium glutamicum* | β-subunit | ATGAGCAATACCACTACCGCGGAAAAGCTGGCGGACCTGCGTGCCCGTCTGGAAATTGCG AAAGACCCGGGCTCTGAACGTGCGCGTAAAAAACGTGACGAGGAAGGCCGTACCACCCCA CGCCAGCGTATTGATGCACTGCTGGACGCAGGCTCTTTTGTTGAGATCGGTGCGCTGGGT CGTACCCCGGATGAGCCGGATGCACCTTACTCCGACGGCGTAGTCACGGGCTACGGTCGT ATCGACGGTCGCCCGGTTGCGATCTACGCCCACGACAAAACCGTCTACGGTGGTTCTGTT GGCATGACCTTCGGCCGCAAAGTTTCCGAAGTTATGGACATGGCGATCCGTATTGGTTGC CCAGTCATTGGTATCCAAGACTCTGGTGGCGCACGCATTCAAGATGCCGTTACCAGCCTG GCGATGTATTCTGAGATTGCGCGTCGCCAACTCCCTCTGTCTGGTCGCTCTCCGCAGATC TCTATCATGCTGGGTAAGTCTGCCGGTGGTGCGGTTTATGCCCCGGTTACCACTGACTTC GTAATTGGCGTTGATGGTGAAACCGAGATGTACGTTACGGGTCCGGCTGTTATCAAGGAA GTTACCGGCGAGCAGATTACTTCTGCCGATCTGGGTGGCGGCGCACAGCAAATGCAAAAC GGCAACATCTCTTACCTGGCGTCTTCTGAAGAGGAAGCCCTGAACATGGTTAAGGATCTC CTCGACTTCCTGCCGCTCACCTGCAATGACCCGGCTCCGGTTTTCGCTGCGCCGACCGAT GAGGAAATCGCCTACGACGAAGCGCTCAACAGCTTCATGCCGGACGACACCAACCAGGGT TACGACATGCACGACCTCCTGGATAAGCTCTTTGACGACGCTAACCTGCTCGAGATTCAG GAAGAATACGCCCCGAACCTGATCACCACCTTCGCCCGTGTTGACGGCAAAGCTGTAGGT GTTGTTGCGAACCAACCGATGGATAAAGCGGGCTGTATTGACGCGGATGCGGCTGATAAA GGTGCTCGTTTCATCCGCATCTGCGACGCATACAACATCCCGATCATCTTCGTTGTTGAC ACCCCGGGTTACCTGCCGGGTGTGGACCAGGAAAAAGTTGGTCTGATTCATCGTGGCGCG AAACTGGCGTTCGCGGTGGTAGAATCTACCGTACCGAAAATCTCTCTCATCGTTCGTAAA GCGTACGGCGGTGCCTACGCGGTTATGGGTTCTAAAAACCTGACCGGTGATCTGAACTTC GCGTGGCCAACTGCGCAAATCGCGGTCATGGGTGCGGCAGCAGCGGTTGTAATGATCCAG GGTAAGCAGCTCGAAGCGGCACCTCCAGAACAACGCGAGTACATGAAGAAACTGTTCATG GACTTCTATGACGAAAACATGACGTCCCCGTATGTTGCTGCGGAACGCGGCTATATCGAT GCCATGATCGAACCGGCGGAAACCCGCCTGGTTCTCCGTCGCGCAGTGCGTCAGCTGGAA ACGAAAGCGGTACGTGACCTCGATAAAAAGCACACCATTATGCCGATG |
| *Danio rerio* | β-subunit | ATGGCGACCTACAAGCTGATGCGTAGCTCTCTCGGTCTGCTCAACGGTCTGAAATGCCCA TTCAAGTGCGTTGGTCAAACCCAGTATGGCGTGTTCACTCAGTCTAAGGTACTGCAGAAT GCCCGCTGCTACTCTTCTTCTCACATGTCTGTTCAAGAGCGCATCGAGCAAAAGCGTCAA GCAGCACTGACGGGCGGTGGCCAACTCCGTATTGCGGCTCAGCACAAACGTGGTAAACTG ACCGCACGTGAGCGTGTTGAACTCCTCCTGGACGCGGACAGCTTTGTGGAATACGACATG TTCGTTGAACACCGTTGCTCTGACTTCGGTATGGAGGCCGATCAGAACAAATATCCGGGT GACTCTGTTGTTACTGGTCAGGGCCGTATTAATGGCCGTCTGGTATACGTTTTCTCCCAG GACTTTACGGTTTTCGGCGGTAGCCTGTCCGGCGCACACGCGCAGAAGATCTGTAAAATC ATGGACCAGGCGATGCTGGTCGGTGCCCCTGTTATTGGCCTGAACGATTCTGGCGGTGCA CGTATTCAGGAGGGTGTTGAGTCTCTCGCGGGTTATGCGGACATTTTCCTCCGTAATGTT ATGGCATCTGGTGTTGTACCGCAGATTTCTCTCATCATGGGTCCGTGTGCAGGCGGCGCA GTGTACAGCCCAGCGCTCACCGACTTCACCTTTATGGTTAAGGACACGAGCTACCTCTTC ATCACGGGCCCAGATGTGGTCAAGTCTGTTACGAATGAGGACGTTACCCAGGAAGAACTC GGTGGTGCAAAGACCCATACGGCTGTTTCCGGTGTTGCACATCGCGCCTTCGAGAACGAT ATTGACGCGCTCCTCAACCTGCGTGACTTCTTCAACTTCCTGCCGCTCTCTAACAAAGAC TCTGCGCCAGTTGTAGAGTGTCACGACCCGCGTGACCGCCTCGTTCCTGGTCTGGACACC GTCGTTCCGTTCGAGTCCACGAAAGCGTACGATATGCTCGACATCGTACACGGCATCGTT GACGAGCGCGAATTTTTCGAGATCATGCCGAACTACGCGAAAAACATTGTCGTGGGCTTT GCCCGTATGAATGGTCGTACCGTGGGTATTGTAGGCAACCAGCCTAAAGTTGCTAGCGGC TGCCTGGACATCAACTCTTCCGTAAAAGGTGCGCGTTTCGTTCGTTTCTGCGACGCCTTT AACATCCCGATCATTACCTTTGTCGATGTTCCGGGCTTCCTCCCGGGTACGGCGCAAGAG TATGGTGGCATCATCCGTCACGGTGCGAAACTGCTGTACGCGTTCGCCGAAGCCACGGTT CCGAAAATCACGGTTATCACCCGCAAGGCCTACGGTGGCGCGTATGACGTTATGTCTTCT AAACACCTGCGCGGTGATGTCAATTACGCGTGGCCGACGGCAGAGGTCGCGGTTATGGGT GCCAAGGGCGCTGTCCAAATCATCTTTCGCGGCAAACAGAACCAGGCTGAAGCAGAGGCA GAGTACGTTGAAAAGTTTGCGAATCCGTTCCCGGCAGCAGTGCGTGGTTTTGTAGATGAT ATTATCCAGCCATCCACCACCCGCCGTCGCATTTGCCGTGACCTGGAAGTTCTCGCGTCT AAAAAACAAACGAATCCATGCTTCCGCGCGTTCCATAATTCCATTTGTGGT |
| *Homo sapiens* | β-subunit | ATGGCAGCGGCGCTGCGTGTTGCGGCAGTGGGTGCTCGTCTCTCTGTTCTCGCCTCTGGC CTCCGTGCGGCCGTACGTAGCCTGTGCTCCCAGGCGACGTCTGTGAACGAACGCATTGAG AATAAGCGTCGTACTGCCCTCCTGGGTGGTGGTCAACGCCGTATTGACGCTCAGCACAAA CGTGGTAAACTGACCGCGCGTGAACGTATTTCTCTGCTCCTGGACCCAGGCTCTTTTGTT GAGTCCGACATGTTCGTTGAACACCGCTGCGCGGACTTCGGTATGGCCGCTGACAAAAAC AAATTCCCGGGTGACTCTGTTGTTACCGGCCGTGGCCGCATTAACGGTCGTCTCGTGTAC GTGTTCAGCCAACAAATCATCGGTTGGGCGCAGTGGCTCCCACTGGTCATCTCTGCGCTG TGGGAAGCGGAAGACTTCACGGTATTTGGTGGTTCCCTGTCTGGCGCGCACGCGCAGAAG ATCTGCAAGATCATGGATCAGGCGATCACTGTTGGTGCGCCGGTTATCGGTCTGAATGAC TCCGGTGGCGCTCGCATCCAGGAAGGTGTTGAATCCCTGGCGGGTTACGCGGATATCTTC CTCCGCAACGTAACGGCTTCCGGTGTAATCCCGCAAATCTCTCTCATCATGGGTCCGTGT GCAGGCGGTGCTGTTTACTCTCCGGCCCTGACCGACTTTACCTTTATGGTGAAGGACACC AGCTATCTGTTTATCACTGGCCCGGACGTGGTTAAGAGCGTAACGAATGAGGACGTCACC CAAGAGGAGCTCGGCGGCGCCAAGACTCATACCACCATGTCTGGTGTTGCTCATCGCGCC TTCGAGAACGATGTGGATGCCCTCTGCAACCTGCGCGACTTCTTCAACTACCTGCCGCTC TCTTCCCAGGACCCGGCACCGGTCCGCGAATGCCACGACCCGTCTGACCGTCTGGTCCCG GAACTCGACACCATTGTTCCACTCGAAAGCACCAAAGCGTACAACATGGTCGACATTATC CATTCTGTGGTAGACGAACGTGAGTTCTTCGAAATCATGCCGAACTATGCAAAGAACATC ATCGTGGGCTTCGCCCGTATGAATGGTCGCACGGTGGGTATCGTTGGTAACCAGCCGAAG GTTGCGTCCGGTTGCCTGGACATCAACTCCAGCGTTAAAGGTGCGCGTTTCGTACGTTTC TGCGACGCGTTCAACATTCCTCTGATTACTTTTGTGGACGTTCCTGGCTTCCTGCCAGGT ACCGCGCAAGAGTACGGCGGTATTATTCGTCACGGTGCCAAGCTGCTGTATGCTTTTGCC GAGGCTACCGTTCCGAAAGTAACCGTAATCACCCGTAAGGCGTACGGTGGTGCCTACGAC GTCATGTCTAGCAAACACCTGTGCGGTGACACCAACTACGCCTGGCCGACCGCGGAAATC GCCGTGATGGGTGCAAAGGGTGCCGTGGAGATCATCTTCAAAGGTCACGAGAATGTGGAG GCGGCTCAGGCGGAATACATCGAAAAGTTCGCCAACCCGTTCCCGGCTGCGGTTCGTGGC TTTGTCGACGATATCATCCAGCCGTCCAGCACTCGTGCCCGCATTTGCTGCGATCTCGAC GTACTCGCTTCTAAAAAAGTACAGCGTCCGTGGCGTAAACACGCGAATATCCCGCTC |
| *Methylobacterium extorquens* | β-subunit | ATGAAAGACATCCTGGAAAAACTCGAAGAACGTCGTGCGCAAGCACGTCTCGGTGGTGGC GAGAAGCGTCTGGAAGCGCAGCACAAGCGTGGCAAGCTGACCGCGCGTGAACGTATCGAA CTGCTGCTGGACCACGGTTCTTTCGAGGAGTTCGACATGTTCGTTCAACACCGTTCTACC GACTTTGGCATGGAGAAACAAAAGATCCCAGGTGACGGTGTGGTGACCGGTTGGGGCACG GTCAATGGTCGCACGGTATTTCTCTTCAGCAAGGATTTTACCGTCTTCGGTGGCAGCCTG TCCGAAGCACACGCAGCCAAGATCGTAAAAGTTCAGGACATGGCGCTGAAAATGCGTGCT CCGATCATCGGTATCTTCGACGCAGGCGGTGCGCGTATCCAGGAAGGCGTTGCGGCGCTG GGTGGTTACGGCGAGGTTTTTCGTCGTAACGTCGCGGCAAGCGGTGTTATCCCGCAGATC TCTGTTATCATGGGCCCGTGTGCGGGTGGCGATGTTTACTCTCCTGCCATGACGGACTTC ATCTTTATGGTACGTGATACCTCCTATATGTTTGTGACTGGCCCGGATGTAGTGAAAACT GTTACCAACGAGGTTGTCACGGCAGAGGAACTCGGCGGCGCCAAGGTTCACACTAGCAAG TCCTCTATCGCAGACGGTTCCTTCGAGAATGATGTAGAAGCGATCCTGCAGATCCGTCGT CTGCTCGACTTCCTGCCGGCCAATAACATCGAAGGTGTGCCGGAAATTGAGTCTTTTGAC GACGTAAACCGCCTCGACAAATCCCTGGACACCCTGATCCCGGACAACCCGAATAAACCA TACGACATGGGTGAACTGATCCGCCGTGTTGTTGATGAGGGCGATTTCTTTGAGATCCAA GCCGCTTACGCCCGTAATATCATTACCGGCTTTGGTCGTGTTGAGGGTCGTACCGTTGGT TTTGTTGCGAACCAACCTCTGGTCCTGGCGGGTGTTCTGGACTCTGACGCATCTCGTAAA GCAGCCCGTTTCGTACGTTTCTGTAACGCGTTTTCTATCCCGATCGTCACTTTCGTAGAT GTTCCAGGTTTCCTCCCGGGTACCGCGCAAGAATACGGTGGCCTGATCAAACACGGTGCC AAGCTCCTGTTCGCGTACTCTCAGGCTACTGTACCACTCGTTACTATTATTACTCGCAAG GCTTTTGGTGGTGCATATGACGTTATGGCGTCTAAACACGTTGGTGCAGACCTCAATTAT GCGTGGCCTACGGCGCAAATCGCGGTTATGGGTGCTAAAGGTGCGGTTGAAATCATCTTC CGTGCGGAAATCGGTGATGCCGACAAAATTGCGGAACGCACCAAGGAGTACGAGGACCGC TTTCTGTCCCCGTTTGTGGCCGCTGAACGCGGCTATATCGATGAGGTAATCATGCCGCAC AGCACGCGTAAACGTATTGCTCGTGCCCTCGGCATGCTCCGTACTAAAGAGATGGAACAG CCATGGAAGAAGCACGACAATATCCCTCTC |
| *Myxococcus fulvus* | β-subunit | ATGGACCAGACCCCGGAGAATGATCCACTCCGTGCGCGCCTGGAGAAAATGGAGAAGCAG GCGGAACTGGGTGGTGGTGCAGACCGCATCGCGAAACAGCACGAAGCCGGCAAGCTGACC GCACGCGAACGTATTGACCTGCTGCTGGACGCGGGTTCTTTCTGCGAGCTGGACAAGTTC GTGACGCACCGTTCTAATGAATTTGGCATGGGCGATAAGAAGATCCCGGGTGACGGTGTA GTAACGGGCTATGGTACGGTCGAAGGTCGTAAAGTTTTCGTGTTCGCACAGGACTTCACC GTCTTTGGCGGTTCTCTGTCTGGCGCTTACGCCCAGAAAATCTGCAAAATCATGGACCTC GCGACTCGCGTAGGTGCCCCGGTCATCGGTCTGAACGATTCTGGCGGCGCACGCATCCAG GAGGGCGTAGAGTCTCTCGCTGGCTACGCCGACATCTTCGTTCGTAATACCCTGGCGAGC GGTGTCGTTCCGCAGATCTCTCTGATCATGGGTCCTTGCGCGGGTGGCGCCGTGTACTCT CCTGCGATTACCGACTTCATCATGATGGTGAAGGACACGAGCTACATGTTTATCACCGGT CCGGATGTTATCAAAACCGTTACCCACGAAGAGGTATCTAAGGAAGCGCTCGGTGGCGCA GTCACTCATAACCAGAAATCCGGTGTTGCACACTTCGCTGCCGAAAACGAACAGGCGGCA ATTGTGATGACGCGTGAACTGCTCTCTTTCCTGCCGTCTAACAATCAGGAAGAAGCGCCT GTGCAGCCGTGCGAAGACGATCCATTCCGTGCCGAAGAATCTCTCAAGACCATCGTACCT GCGAACCCGAACAAACCGTACGACATCAAGGAGGTCATCAAAGCGATCGTGGACGACAAA CACTTCTTCGAGGTTCAGGAGCACTTTGCCAAGAACATCGTCATTGGCTTCGCGCGCATG AATGGTCGTTCTGTTGGCGTAGTCGCAAACCAGCCAGCTGTCCTGGCAGGCGTTCTCGAC ATTGACGCCTCCATTAAAGCAGCGCGTTTCGTACGTTTCTGTGACTGTTTCAACATCCCT CTGGTGACGCTGGTTGACGTGCCGGGCTTCCTCCCTGGTACCGACCAGGAATGGGGTGGT ATCATTACGCATGGCGCTAAGCTCCTCTATGCCTACGCAGAAGCGACGGTTCCAAAGGTT ACCGTTATTACCCGCAAAGCATACGGCGGTGCATATGACGTTATGGCCAGCAAACACATC CGTGCTGACATGAACTTTGCGTGGCCGACCGCGGAAATCGCCGTGATGGGCCCGGAGGGT GCCGTTAATATCATCTTCCGCAATGAGCTGCTGAAAGCGAAAGACGCTGCGGCGGAGCGT GCTAAACTCACCGCAGACTACCGCGAAAAGTTCGCGACCCCGTTTAAGGCGGCTGAACTC GGCTACATCGACGAGATCATCCGCCCGGAAGAAACCCGTGCAAAACTGATTCGTGCCCTG GAACTCCTCAAAGACAAACGTCAGGAAAACCTGCCACGTAAACACGGTAATATCCCGCTG |
| *Paracoccus sp.* | β-subunit | ATGAAGCTCGGTACTTCCGTGCTGCCTTCTTCCGATAACTTTCGTGCGAATCGCGAGGCT CATCTCGCAATGCTGGAAACCGTACGCGAAGCGGCGCTGGCGGCAGCCGCAGGTGGCGGT CCTAAGGCGATGGAACGTCACACCTCTCGTGGTAAAATGCCTCCACGTGAACGTGTAGCG AACCTCCTCGACCCTGGCAGCCCGTTCCTGGAGATCGGTGCGACCGCGGCTCACGGTATG TACGACGGTGCCGCTCCGTGTGCGGGTGTTATCGCGGGCATTGGCCGTGTTCACGGTCAA GAAGTTATGGTTGTTGCGAATGACGCGACGGTTAAAGGCGGCACGTATTACCCGATGACC GTTAAGAAGCACCTCCGCGCTCAGGAAATTGCGGAGGAGTGTCACCTCCCTTGCGTCTAC CTGGTAGACTCTGGCGGTGCCAACCTCCCGAACCAAGATGAAGTTTTCCCGGATCGTGAC CACTTCGGTCGTATCTTTTACAACCAGGCCCGTATGAGCGCCAAAGGTATTGCGCAGATT GCGGTTGTGATGGGTTCTTGCACCGCGGGTGGCGCCTACGTTCCGGCGATGAGCGATGTG ACCATCATCGTACGTAACCAGGGTACCATCTTTCTCGCCGGTCCGCCGCTCGTAAAAGCA GCGACGGGTGAAGTTGTATCTGCTGAAGACCTCGGTGGTGGCGACGTTCATACCCGTCTG AGCGGTGTCGCGGATTACCTGGCGGAAGACGACGCGCATGCACTCGCCATTGCACGTCGC TCTATCGCCAATCTGAATCGCACCCAGCCAGTTTCCGTTCAGTGGCAGTCTTCTGAACCA CCGGCGTATGACCCGGACGAAATCCTCGGTATCGTCCCGGCAGACCTCAAAATCCCGTAC GACATCCGTGAAGTCATTGCCCGTGTGGCGGATGGCTCTCGTTTCGACGAATTCAAGGCA CGTTTCGGCGAAACCCTCGTAACTGGTTTTGCGCATGTTGAAGGTTGCCCGGTTGGTATC ATCGCGAATAACGGCGTTATCTTCTCCGAAGCCGCGCAGAAGGGTGCCCATTTTATCGAG CTCTGCTCTATGCGTGGTATCCCACTGGTGTTCCTCCAGAACGTTACGGGCTTCATGGTG GGCCGTAAATACGAAAACGAAGGTATCGCACGCCACGGCGCGAAAATGGTAACGGCTGTT GCTACGACGTCTGTTCCAAAAATCACGATGCTCGTCGGTGGCAGCTTCGGCGCAGGCAAT TACGGCATGTCCGGTCGCGCATATTCCCCACGCTTCCTGTGGACTTGGCCTAATAGCCGC ATCTCTGTCATGGGTGGTGAACAGGCGGCTGGCGTCCTGGCTACCGTCCGTCGTGAGGGT ATCGAACGTGAAGGTGGCACGTGGTCTGCAGGTGAAGAGGCGGAGTTCAAACGCCCAACC ATCGAAATGTTCGAGCGTCAGTCTCACCCACTGTACGCGTCCGCACGTCTCTGGGACGAC GGCATTATTGACCCGCGTAAAACCCGCGAAGTACTGGCACTGTCCCTCCGTGCGAGCCTG AATGCTCCGGTTGAACCAACGCGCTTTGGCATCTTCCGTATG |
| *Propionibacterium acnes* | β-subunit | ATGGAGATCGATATCCACACGACTGCGGGTAAAATTGCGGATCTGGGTCGCCGTATTGAC GAAGCGGTTAACGCCGCGTCTCCATCTGCGATCGAGAAACAACATGCGACCGGTAAGATG ACTGCGCGTGAACGTATCCTCCGCCTCCTGGATGAGGATTCCTTTACCGAACTCGACGAG TTTGCTCGTCACCGTAGCACTAATTTTGGCATGGATCGCAAGCGCCCGTATACCGACGGC GTGATTACTGGTGTTGGTGCGATTCATGGCCGTCCGGTTTGCGTCTTTAGCCAAGACGTA ACCATTTTCGGTGGCTCCCTCGGTGAGGTGTATGGCGAGAAAATCTGCAAAATCATCGAC TTCGCGGTCAAGACCGGTTGCCCGCTGATCGGCATTAACGAGGGTGGTGGTGCACGCATT CAGGAGGGTGTTGCCTCTCTGGCTCGTTTTGGCGATATTTTCCGTCGCAACACTCGTGCG AGCGGTGTTATTCCGCAGATCTCTATCATTATGGGCGCAGCAGCCGGTGGTCACGTGTAC TCTCCTGCGCTGACCGACTTCATTGTGATGGTGGACCAGACGTCCCAGATGTTCATCACT GGTCCGGCGGTAGTTAAGCAGGTTACGGGTGAGGACGTTTCTCTGGAGGAACTGGGCGGT GCGCGTACCCACAGCGTTAAGAGCGGTAACAGCCACTACCTGGCCAACGATGAAGACGAC GCCCTGGAGTTCGTCCGTGACCTGATTTCCTACCTCCCACAGAACAACCTCGAAGACCCG CCTTTCTACGACGATGGCGAAGCTGATCTCACGATCACCGACCATGACCGCAAGCTGGAT GTTCTGATCCCGGACTCTTCTCACCAGCCGTACGATATGCATGAGGTCATCACGACCGTT CTGGACGAAGACACTTTCCTGGAAATCCACGAGCTGTTCGCGCCGAACGTTATTTGCGGC TTTGGCCGTATCGAAGGTCGTGTCATTGGTATCGTTGCGAATCAGCCGATGGTCAACGCA GGCACCCTGGACATTGACGCCTCCGAAAAGGCAGCGCGTTTTGTTCGCACTTGCGACTCT TTCAACATTCCGGTTCTCACGTTCGTTGATACCCCGGGTTTCCTGCCGGGTGTCGAGCAG GAACACGATGGTATTATTCGCCGTGGTGCCAAACTCATTTATGCTTACGCCGAAGCAACG GTTCCGCTGCTGACGGTAGTGACGCGTAAGGCGTACGGTGGCGCGTATATCGTCATGGGC TCTAAAACGCTCGGTGCCGACGTAAATCTCGCGTGGCCGACCGCGCAGATTGCGGTGATG GGTGCTGAAGGTGCGGTGTCTATCCTGCACCGTCGTACCCTCGCGACCGACCCGGATCCA CAGGCGAAACGCAAAGAACTGATTGATGAGTACGAAACGACGCTGTCTAACCCATACCAG GCTGCAGAGCGTGGTTGGATTGACCAAGTCATTCACCCGCATGAAACGCGTGCGTCTGTC ATCCGCACTCTCCGTCTGCTCCGTACCAAGCGTGAAGCTCTCCCGCCTAAGAAACACGGC AACATCCCGCTG |
| *Rhodobacter sphaeroides* | β-subunit | ATGAAAGATATCCTCCAAGAACTCGAGAATCGCCGTGCTATCGCTCGCGCAGGCGGTGGT CAGCGTCGCGTTGAAGCACAACACAAACGTGGTAAACTGACCGCGCGTGAACGTATTGAG CTCCTGCTCGACGAAGGCTCCTTTGAAGAGTTCGATATGTTTGTGCGTCACCGTTGCACC GATTTTGGTATGCAGGATGACCGTCCAGCCGGCGATGGCGTTGTTACCGGTTGGGGTACC ATCAACGGTCGTATGGTTTACGTTTTCAGCCAGGACTTTACCGTCTTCGGTGGTTCCCTG TCCGAAACCCATGCGCAGAAAATCTGCAAAATCATGGACATGGCGATGCAAAACGGTGCT CCAGTTATTGGCCTGAATGACTCCGGTGGCGCACGCATCCAGGAGGGCGTTGCGTCCCTC GCGGGTTATGCCGATGTCTTCCAGCGCAACATTATGGCTAGCGGCGTGATCCCACAGATC TCTGTTATCATGGGCCCTTGTGCAGGTGGCGCTGTATATTCTCCGGCCATGACGGATTTT ATCTTCATGGTTCGTGACACCTCTTACATGTTCGTTACGGGCCCTGACGTCGTGAAAACT GTGACCAACGAAGTGGTAACCGCCGAGGAGCTGGGTGGTGCATCCACCCACACCAAAAAA TCTTCTGTCGCCGACGGTGCATTCGAAAACGATGTCGAAGCGCTCTATGAGATCCGTCGC CTGGTCGACTTTCTCCCGCTCTCTAACCGCACTCCGGCACCGGTTCGTCCATTCTTTGAC GACGTTGCGCGTATCGAGGATTCTCTGGACACCCTGATCCCGGACAACCCGAACCAGCCG TACGACATGAAGGAACTGATTCTGAAAATCGCGGACGAAGCCGACTTCTACGAGATCCAA AAGGACTTCGCGGCCAACATCATTACTGGTTTCATTCGTCTGGAGGGCCAGACTGTTGGT GTTGTTGCAAACCAACCAATGGTGCTGGCCGGTTGCCTGGACATCGACTCCTCTCGCAAG GCTGCCCGTTTCGTTCGCTTCTGTGACGCGTTCAACATCCCGATCCTGACTCTCGTTGAC GTGCCGGGTTTCCTGCCTGGTACTGGCCAAGAGTACGGCGGCGTTATCAAACATGGTGCG AAACTGCTGTTCGCGTACGGTGAGGCGACCGTACCGAAAGTTACGGTTATCACCCGCAAA GCTTATGGCGGTGCGTATGACGTAATGGCGAGCAAGCACCTGCGTGGCGACTTCAACTAC GCTTGGCCGACTGCCGAGATCGCGGTTATGGGTGCTAAAGGTGCGACCGAAATCCTGTAC CGTTCTGAACTCGGTGACAAGGAGAAAATTGCAGCCCGTGCCAAGGAATACGAAGATCGC TTTGCCAACCCGTTTGTAGCCGCGGAACGTGGCTTCATTGACGAAGTAATCATGCCGCAT TCTACCCGTCGTCGTGTTTCTAAAGCGTTCGCGAGCCTGCGTAACAAAAAACTGGCGAAC CCGTGGAAAAAGCACGATAACATTCCACTG |
| *Sinorhizobium fredii* | β-subunit | ATGCGTGCGATCCTCGAACAAGTAGAAGCGCGTCGTGCGCAGGCACGCGCTGGTGGTGGC GAGCGTCGTATTGCAGCGCAACATGGTAAAGGTAAACTCACCGCGCGTGAGCGCATTGAC GTACTGCTGGACGAGGGCTCTTTCGAAGAATACGATATGTACGTGACCCACCGTTGCGTC GACTTCGGTATGGCCGAAGAAAAGATTGCGGGTGATGGCGTTGTTACCGGTTGGGGTACG ATTAACGGTCGTCAGGTGTATGTGTTCTCTCAGGACTTCACTGTACTGGGCGGTTCTCTG TCTGAAACCCACGCGCAAAAGATCTGCAAGATCATGGATATGGCTGCACGTAACGGTGCG CCGGTGATTGGTCTGAATGATAGCGGTGGCGCACGTATCCAGGAAGGTGTTTCTTCTCTG GCCGGTTATGCAGAAGTTTTCCGTCGTAACGCTGAAGTAAGCGGCGTTATCCCGCAGATT TCCGTAATTATGGGTCCGTGTGCAGGTGGCGCTGTCTACTCTCCGGCGATGACGGATTTC ATCTTCATGGTTCGTGACTCTTCTTACATGTTCGTAACCGGTCCTGACGTTGTAAAAACC GTGACCAACGAAATCGTTACCGCGGAGGAGCTCGGTGGTGCACGCACTCACACGACCAAA TCTAGCGTTGCGGATGGTGCGTATGAGAACGACGTTGAAGCGCTGGAACAGGTTCGCCTG CTGTTCGATTTCCTGCCGCTGAACAATCGCGAGAAACCGCCGGTTCGTCCATTTCATGAT GACCCTGCCCGTCTGGAGATGCGTCTCGACTCTCTGATCCCGGAAAGCGCGGCAAAACCT TACGACATGAAAGAACTCATTCTGGCAGTAGCAGACGAAGGTGACTTCTTTGAACTGCAG GCGGGCTTTGCTCGTAACATCATTACTGGTTTCATCCGCCTGGAGGGCCAAACCGTAGGT GTGGTTGCGAACCAGCCAATGGTGCTCGCAGGCTGTCTGGACATCGATTCCTCCCGTAAA GCGGCACGTTTCGTTCGTTTCTGCGACGCGTTCTCTATCCCAATCCTCACGCTCGTTGAT GTACCGGGTTTTCTGCCTGGTACCGCACAGGAATACGGCGGCGTGATCAAACACGGCGCG AAACTCCTCTTTGCGTACTCTCAAGCAACCGTTCCGATGGTGACGCTGATTACCCGTAAG GCGTATGGCGGTGCTTACGATGTTATGGCGTCTAAACACATCGGTGCGGACGTGAACTAT GCCTGGCCGACTGCGGAAATTGCGGTCATGGGTGCAAAAGGTGCAACCGAAATCCTCTAC CGTTCTGAACTCGGCGACCCGGAGAAAATCGCGGCTCGTACCAAAGAATATGAAGAGCGT TTTGCGAATCCGTTTGTTGCGGCGGAACGTGGCTTCATCGACGAAGTTATCATGCCGCAT AGCTCTCGCCGTCGCATTGCCCGTGCGTTCGCGAGCCTCCGTAACAAGCAGGTTGAGACT CGCTGGCGCAAACATGACACGATCCCGCTG |
| *Streptomyces coelicolor* | β-subunit | ATGTCTGAGCCTGAAGAACAACAGCCGGACATTCACACCACTGCAGGTAAGCTCGCCGAT CTCCGTCGTCGTATTGAGGAGGCGACTCATGCGGGTTCTGCACGCGCAGTGGAAAAACAG CATGCGAAAGGTAAACTCACGGCCCGTGAACGTATCGACCTGCTGCTCGATGAGGGTAGC TTCGTTGAACTGGACGAGTTCGCTCGCCATCGCAGCACTAACTTCGGTCTGGACGCGAAC CGTCCTTATGGCGACGGCGTAGTTACCGGCTACGGTACTGTTGATGGCCGTCCGGTTGCT GTGTTCTCTCAAGACTTTACCGTGTTTGGTGGTGCACTGGGTGAGGTGTACGGCCAGAAG ATCGTCAAAGTTATGGACTTCGCGCTGAAAACGGGCTGCCCGGTTGTAGGTATCAATGAC TCCGGTGGTGCTCGCATCCAGGAAGGCGTTGCATCTCTCGGTGCGTACGGTGAGATTTTC CGCCGTAACACGCACGCATCTGGCGTGATCCCGCAAATTTCCCTCGTAGTTGGTCCGTGC GCTGGCGGCGCTGTATATTCTCCAGCCATCACCGACTTCACCGTGATGGTTGACCAGACC TCTCACATGTTCATTACTGGCCCGGATGTAATCAAAACGGTAACCGGTGAGGACGTCGGT TTCGAAGAACTGGGCGGTGCCCGCACCCACAATTCTACCTCTGGTGTTGCGCATCACATG GCCGGTGACGAGAAAGACGCTGTTGAATATGTTAAACAGCTGCTGTCTTATCTGCCGTCC AACAACCTGTCTGAACCGCCTGCGTTCCCGGAAGAAGCCGACCTCGCAGTCACGGACGAA GATGCGGAACTCGACACCATCGTTCCGGACTCTGCGAACCAGCCGTATGACATGCACAGC GTTATCGAACACGTACTGGATGATGCCGAATTTTTCGAGACGCAGCCGCTGTTCGCGCCG AACATTCTCACGGGTTTTGGTCGTGTCGAAGGTCGCCCTGTTGGCATTGTTGCGAATCAG CCGATGCAGTTTGCTGGCTGTCTGGATATCACGGCGTCTGAGAAAGCAGCGCGTTTCGTT CGTACCTGTGACGCGTTTAACGTGCCGGTTCTCACTTTCGTCGATGTGCCGGGCTTCCTG CCTGGCGTAGACCAAGAACATGACGGTATCATTCGTCGTGGTGCGAAACTCATCTTTGCT TACGCGGAAGCGACCGTACCGCTCATCACTGTTATCACTCGCAAGGCATTCGGTGGCGCT TACGACGTTATGGGCTCTAAACACCTGGGCGCAGATCTCAATCTCGCGTGGCCGACGGCC CAAATTGCGGTAATGGGTGCACAGGGTGCAGTCAACATCCTCCACCGTCGCACTATCGCG GACGCGGGTGATGACGCGGAGGCCACCCGTGCCCGTCTGATTCAGGAATACGAGGACGCG CTCCTGAACCCGTATACTGCCGCGGAGCGTGGTTACGTTGATGCGGTGATCATGCCTTCT GACACTCGTCGTCATATCGTACGCGGCCTGCGCCAACTGCGCACCAAGCGTGAATCTCTG CCTCCGAAAAAGCATGGTAATATCCCACTG |

## Table S4. Genetic element sequences used in this study.

| **Genetic element** | **DNA sequence** |
| --- | --- |
| T7 promoter | TAATACGACTCACTATAGG |
| lac operator | GGAATTGTGAGCGGATAACAATTCCCC |
| 5’UTR-B | GGGGAATTGTGAGCGGATAACAATTCCCCTCTAGAAATAATTTTGTTTAACTTTAAGAAGGAGATATACC |
| N-terminal 6-His tag of beta subunit | ATGGGCAGCAGCCATCATCATCATCATCACAGCAGCGGCCTG |
| C-terminal Flag tag of beta subunit | TCTTCTGGTCTGGTTCCGGACTACAAAGATGATGACGACAAG |
| 5’UTR-A | TAAATAATTTAAAAAACAGACCTCATATCGAAATAAAAGAAGGAGATATACC |
| N-terminal Flag tag of alpha subunit | ATGGGCAGCGACTATAAAGACGACGATGACAAATCTTCCGGC |
| C-terminal 6-His tag of alpha subunit | AGCAGCGGCCTGGTGCCGCGCGGCCATCATCATCATCATCAC |
| T7 terminator | TAAGATCCGGCTGCTAACAAAGCCCGAAAGGAAGCTGAGTTGGCTGCTGCCACCGCTGAGCAATAACTAGCATAACCCCTTGGGGCCTCTAAACGGGTCTTGAG |

## Table S5. Kinetic parameters of PCC complexes and 6-dEB titers of the corresponding E. coli strains. *M. xanthus PCC Km was measured.

| **PCC** | **6-dEB titer [mg/l]** | **PCC expression host** | **Km [uM]** | **Reference** |
| --- | --- | --- | --- | --- |
| *M. fulvus** | 6 | *M.xanthus* | 32 | 10 |
| *C. glutamicum* | 4 | *C.glutamicum* | 97 ± 17 | 11 |
| *S. coelicolor* | 1.4 | *S.coelicolor* | 76.5 ± 5.5 | 12 |
| *H. sapiens* | 0.4 | *E. coli* | 410 ± 0.06 | 13 |

# Supplementary References

1. Pfeifer, B. A., Admiraal, S. J. & Gramajo, H. Biosynthesis of complex polyketides in a metabolically engineered strain of *E. coli*. *Science* **291,** 1790–1792 (2001).

2. Hoover, D. M. & Lubkowski, J. DNAWorks: an automated method for designing oligonucleotides for PCR-based gene synthesis. *Nucleic Acids Res.* **30,** e43 (2002).

3. Gibson, D. G. *et al.* Enzymatic assembly of DNA molecules up to several hundred kilobases. *Nat. Methods* **6,** 343–345 (2009).

4. Dower, W. J., Miller, J. F. & Ragsdale, C. W. High efficiency transformation of *E. coli* by high voltage electroporation. *Nucleic Acids Res.* **16,** 6127–6145 (1998).

5. Lowry, B. *et al.* *In vitro* reconstitution and analysis of the 6-deoxyerythronolide B synthase. *J. Am. Chem. Soc.* **135,** 16809–16812 (2013).

6. Bradford, M. M. A rapid and sensitive method for the quantitation of microgram quantities of protein utilizing the principle of protein-dye binding. *Anal. Biochem.* **72,** 248–254 (1976).

7. Sievers, F. *et al.* Fast, scalable generation of high-quality protein multiple sequence alignments using Clustal Omega. *Mol. Syst. Biol.* **7,** (2011).

8. Stamatakis, A. RAxML Version 8: A tool for phylogenetic analysis and post-analysis of large phylogenies. *Bioinformatics* 1–2 (2014).

9. Jones, D. T., Taylor, W. R. & Thornton, J. M. The rapid generation of mutation data matrices from protein sequences. *Comput. Appl. Biosci.* **8,** 275–282 (1992).

10. Kimura, Y., Kojyo, T., Kimura, I. & Sato, M. Propionyl-CoA carboxylase of *Myxococcus xanthus*: catalytic properties and function in developing cells. *Arch. Microbiol.* **170,** 179–184 (1998).

11. Gande, R. *et al.* The Two Carboxylases of Corynebacterium glutamicum Essential for Fatty Acid and Mycolic Acid Synthesis. *Journal of Bacteriology* **189,** 5257–5264 (2007).

12. Diacovich, L. *et al.* Kinetic and structural analysis of a new group of acyl-CoA carboxylases found in *Streptomyces coelicolor A3(2)*. *J. Biol. Chem.* **277,** 31228–31236 (2002).

13. Jiang, H., Rao, K. S., Yee, V. C. & Kraus, J. P. Characterization of Four Variant Forms of Human Propionyl-CoA Carboxylase Expressed in Escherichia coli. *J. Biol. Chem.* **280,** 27719–27727 (2005).
